# Deficiency of Aging‐Related Gene Chitinase‐Like 4 Impairs Olfactory Epithelium Homeostasis

**DOI:** 10.1111/cpr.70055

**Published:** 2025-05-19

**Authors:** Tingting Wu, Weihao Li, Liujing Zhuang, Jinxia Liu, Ping Wang, Ye Gu, Yongliang Liu, Yiqun Yu

**Affiliations:** ^1^ ENT Institute and Department of Otorhinolaryngology, Eye & ENT Hospital Fudan University Shanghai China; ^2^ Olfactory Disorder Diagnosis and Treatment Center, Eye & ENT Hospital Fudan University Shanghai China; ^3^ Biosensor National Special Laboratory, Key Laboratory for Biomedical Engineering of Education Ministry, Department of Biomedical Engineering Zhejiang University Hangzhou China; ^4^ Department of Otolaryngology Zibo Central Hospital Zibo Shandong China

**Keywords:** aging, Chil4, globose basal cell, homeostasis, olfactory epithelium, olfactory sensory neuron

## Abstract

Mammalian olfactory epithelium (OE) undergoes consistent self‐renewal throughout life. In OE homeostasis, globose basal cells (GBCs) contribute to the generation of olfactory sensory neurons (OSNs) to replace old ones. Chitinase‐like 4 (Chil4), a chitinase‐like protein expressed in supporting cells, plays a critical role in OE regeneration, while its role in tissue homeostasis is still elusive. Here, we found that Chil4 is upregulated in the aged OE. Deletion of Chil4 leads to a reduction in the number of GBCs and immature OSNs (iOSNs). Chil4^−/−^ GBCs show attenuation in cell cycle progression and an aberrant expression pattern of cell‐cycle‐related genes such as Cdk1. Chil4 deletion causes loss of a specific subcluster of GAP43^+^ iOSNs expressing Cebpb, Nqo1 and low level of mature OSN (mOSN) marker Stoml3 (iOSN_CeSt^L^Nq), potentially suggesting a transitional state between immature and mature neurons. Chil4 knockout induces inflammatory activation in Iba1^+^ microglia (MG)‐like cells in the OE. Chil4 downregulation in aged organoids reduced the number of mature sensory neurons, suggesting a necessary role of Chil4 in maintaining neuronal generation in the aged OE. Collectively, these observations reveal a previously unidentified function of Chil4, establishing the cellular mechanism underlying OE homeostasis.

## Graphical abstract

Deficiency of Chil4 impairs OE homeostasis through loss of sustentacular cells and a specific type of immature sensory neuron, cell cycle arrest in globose basal cells, and inflammatory activation in microglia‐like cells.

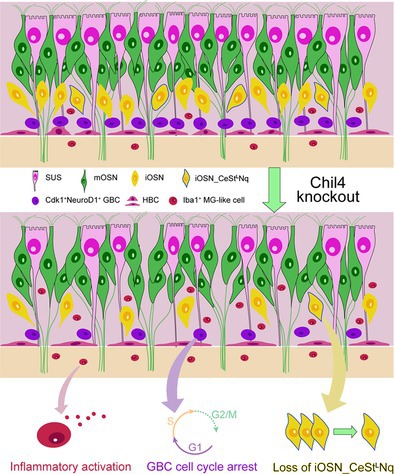

**Abbreviations**
Aif1: allograft inflammatory factor 1
Alox15: arachidonate 15‐lipoxygenase
Arf5: ADP‐ribosylation factor 5
Ascl1: achaete‐scute family bHLH transcription factor 1
Ascl3: achaete‐scute family bHLH transcription factor 3
Bard1: BRCA1 associated RING domain protein 1
BirC5: baculoviral IAP repeat‐containing 5
C3: component 3
CAMk2d: calcium/calmodulin‐dependent protein kinase II delta
Ccl11: C‐C motif chemokine ligand 11
Ccna2: cyclin A2
Ccr2: C‐C chemokine receptor type 2
Cdk1: cyclin‐dependent kinase 1
Cdk4: cyclin‐dependent kinase 4
Cebpb: CCAAT/enhancer binding protein beta
Cfap126: cilia and flagella associated protein 126
Cftr: cystic fibrosis transmembrane conductance regulator
Chil4: Chitinase‐like 4
Cxcl5: C‐X‐C motif chemokine ligand 5
Cyp2g1: cytochrome P450 family 2 subfamily G member 1
Dclk1: doublecortin‐like kinase 1
Dcn: decorin
Dyrk3: dual‐specificity tyrosine‐(Y)‐phosphorylation regulated kinase 3
Efna: ephrin A
Eid1: E1A binding protein p300
Epha: eph receptor A
Ermn: ermin
F4/80: F4/80 antigen, adhesion G protein‐coupled receptor E1
Fcrls: Fc receptor‐like S
Foxj1: forkhead box J1
Gap43: growth‐associated protein 43
GBC: globose basal cell
Gng8: G protein subunit gamma 8
H2‐Ab1: histocompatibility 2, class II antigen A, beta 1
HBC: horizontal basal cell
Hbegf: heparin‐binding EGF‐like growth factor
Hes1: hairy and enhancer of split 1
Hexb: hexosaminidase B
Iba1: induction of brown adipocytes 1
Icam1: intercellular adhesion molecule 1
IL33: interleukin 33
iOSN: immature olfactory sensory neuron
Krt14: keratin 14
Krt19: keratin 19
Krt5: keratin 5
Lcn11: lipocalin 11
Lhx2: LIM homeobox 2
Lum: lumican
MG: microglia
Mgp: matrix Gla protein
mOSN: mature olfactory sensory neuron
MPO: myeloperoxidase
Muc5b: mucin 5B
Mup4: major urinary protein 4
Mup5: major urinary protein 5
Myh11: myosin heavy chain 11
Ncam1: neural cell adhesion molecule 1
Ndfip1: Nedd4 family interacting protein 1
NeuroD1: neurogenic differentiation 1
Neurog1: neurogenin 1
Nqo1: NAD(P)H dehydrogenase, quinone 1
Obp1a: olfactory binding protein 1A
Obp1b: olfactory binding protein 1B
Obp2b: olfactory binding protein 2B
OE: olfactory epithelium
Omp: olfactory marker protein
OSN: olfactory sensory neuron
Pax6: paired box 6
PGP9.5: UCHL1, ubiquitin C‐terminal hydrolase L1
PLCβ2: phospholipase C beta 2
Plena4: plenin 4
Rpl38: ribosomal protein L38
Rps29: ribosomal protein S29
S100a4: S100 calcium binding protein A4
S100a5: S100 calcium binding protein A5
S100b: S100 calcium binding protein B
scRNA‐Seq: Single‐cell RNA sequencing
Sema6a: semaphorin 6A
Sox17: SRY‐box transcription factor 17
Sox2: SRY‐box transcription factor 2
Stoml3: stomatin‐like 3
Sult1c1: sulfotransferase family 1C member 1
SUS: sustentacular cell
Tag1n: taglin
Tmem59: transmembrane protein 59
Trem2: triggering receptor expressed on myeloid cells 2
Trpm5: transient receptor potential cation channel subfamily M member 5
Tuj1: neuron‐specific class III beta‐tubulin
Ugt2a1: UDP glucuronosyltransferase 2 family member A1

## 1 Introduction

The sense of smell is important for nutrition intake, danger recognition, and other biological functions in mammals. This sense largely depends on the olfactory sensory neurons (OSNs) residing in the olfactory epithelium (OE). Neurogenesis occurs in the mammalian OE throughout life, while two types of basal cells contribute to this process [1]. Globose basal cells (GBCs) show proliferative capacity and are responsible for maintaining the OE homeostasis, while horizontal basal cells (HBCs), dormant in the uninjured OE, are recruited for tissue regeneration upon severe injury [2]. However, the neurogenesis in the OE is not unlimited. Factors such as viral infection, tissue damage, and toxic exposure cause severe damage to the OE [3, 4, 5], which may ultimately lead to olfactory dysfunction (OD). Therefore, investigating key genes and their regulatory mechanisms involved in OE homeostasis and regeneration is of great significance for understanding the occurrence and precise intervention of the OD.

A set of regulators such as critical signalling molecules [6] and transcriptional factors [7] control OE homeostasis and regeneration. Chitinase‐like 4 (Chil4, also known as Chi3l4, Ym2) is a subtype of chitinase‐like proteins (CLPs) belonging to the glycoside hydrolase family 18. It has been reported that Chil4 is associated with tissue repair and fibrosis [8, 9], immune regulation [10, 11, 12], pathogen infection [13, 14], and tumour progression [15], making it play an important role in tissue damage and regeneration, chronic inflammatory diseases, host defences, and homeostasis maintenance. Studies have reported an elevated Chil4 expression in the OE after inhalation of *Alternaria* [14] and induction of IL‐13 expression [16]. Our previous study has also shown that Chil4 was upregulated in the sustentacular cells (SUSs) after OE injury and regulated OE regeneration [17]. These results indicate that Chil4 plays a critical role in the OE upon injury. However, how it behaves in OE homeostasis and the underlying molecular mechanism remains elusive. Thus, it is worthwhile to study how Chil4 regulates OE homeostasis, which can provide direct evidence for establishing the regulatory network of basal cell differentiation and tissue regeneration.

Inflammation is crucial in OE homeostasis and regeneration. Our previous work found that the knockout of a key OE homeostasis gene Tmem59 (transmembrane protein 59) activated the inflammatory microenvironment and impaired olfactory function, while anti‐inflammation alleviated neuronal deficits and smell loss caused by gene deletion [18]. In addition, an acute inflammatory microenvironment was necessary for OE regeneration [19], while chronic inflammation blocks the regenerative function of HBCs [20]. Our publicly available single‐cell RNA sequencing (scRNA‐Seq) data revealed a direct interaction between immune/inflammatory cells and HBCs [21]. These results imply that inflammation plays a complex role in regulating olfactory epithelial homeostasis, promoting tissue regeneration, and preserving olfactory function. In our previous study, we found that the overexpression of Chil4 counteracted the attenuation of OE regeneration by anti‐inflammatory treatment, suggesting that Chil4 regulates OE regeneration via interaction with inflammation [17]. Thus, the molecular mechanism underlying the correlation among Chil4, inflammation, and tissue homeostasis needs further investigation.

In the current study, we identified aging‐related elevation of Chil4 and its related genes in the OE, in which arachidonate 15‐lipoxygenase (Alox15) and C‐X‐C motif chemokine ligand 5 (Cxcl5) were reported to be related to inflammatory response and immune regulation [16, 22, 23, 24]. Chil4 deficiency impaired homeostasis of the young OE, with a reduction in the number of GBCs, immature OSNs (iOSNs) and SUSs. Deficiency of Chil4 also led to cell cycle arrest in GBCs and the loss of a specific subset of immature neurons, which were in a transitional state toward mature neurons. In the Chil4^−/−^ OE, inflammation was promoted in Iba1^+^ microglia (MG)‐like cells, with enhanced communication from HBCs to MG‐like cells mediated through the CD44 receptor, which functions in wound healing and inflammatory process [25, 26]. Additionally, anti‐inflammation by dexamethasone (Dex) recovered the homeostasis of immature neurons in the Chil4^−/−^ OE. By using in vitro‐cultured OE organoid models, we found that Chil4 downregulation in the aged organoids impaired neuronal and sustentacular generation, potentially suggesting that higher expression of Chil4 is essential for homeostasis of the aged OE. Collectively, this study describes a critical role of Chil4 in maintaining olfactory epithelial homeostasis.

## 2 Materials and Methods

### 2.1 Animals

Chil4^−/−^ mice were generated by GemPharmatech Corp. (Nanjing, China). The wild type (WT) C57BL/6J mice were purchased from Shanghai Jiesijie Laboratory Animal Co. Ltd. WT and Chil4^−/−^ mice aged 3–4 months were used for single‐cell RNA sequencing, immunostaining, RNAscope, Western blot, in vitro microelectrode array recordings, and organoid culture. Young WT mice aged 3 months and aged WT mice aged 22–24 months were used for single‐cell RNA sequencing, immunostaining, RNAscope, organoid culture, and adeno‐associated virus (AAV) infection. Mice were housed with open access to food and water at the Experimental Animal Facility of Shanghai Medical College, Fudan University (Shanghai, China). The animals were housed in an environment with a 12‐h light/dark cycle. The procedures for animal breeding and tissue harvesting were approved by the Committee of Laboratory Animals in Fudan University (Permit number: SYXK2020‐0032).

### 2.2 Cryosection Preparation and Immunostaining

Mice were deeply anaesthetised before decapitation. Sacrificed mice were transcardially perfused with phosphate buffer saline (PBS) and 4% paraformaldehyde (PFA) before the heads were dissected. The heads were fixed in 4% PFA overnight at 4°C and then decalcified in 0.5 M Ethylenediaminetetraacetic acid (EDTA) for 4–6 days at 4°C. Tissues were then sequentially dehydrated in 10%, 20%, and 30% sucrose and embedded in Tissue‐Tek optimal cutting temperature (O.C.T.) compound. The frozen tissues were cut into 20‐μm coronal sections using a Cryostat (model CM1950, Leica).

Immunostaining was performed according to a standard protocol. After rinsing with PBS three times, the tissue sections were blocked in BSAT (0.3% Triton X‐100 in PBS with 5% bovine serum albumin) at room temperature for 1 h, and then incubated with the primary antibodies at 4°C overnight. The primary antibodies used were as follows: rabbit anti‐Krt14 (#10143–1‐AP, Proteintech), rabbit anti‐Sox2 (#AF‐2018, R&D Systems), goat anti‐Icam1 (#AF796, R&D Systems), rabbit anti‐Chil4 (#ab192029, Abcam), rabbit anti‐OMP (#ab183947, Abcam), mouse anti‐Tuj1 (#ab78078, Abcam), rabbit anti‐GAP43 (#ab75810, Abcam), rabbit anti‐NeuroD1 (#ab205300, Abcam), mouse anti‐Ki67 (#550609, BD Biosciences), goat anti‐IL33 (#AF‐3626, R&D Systems), mouse anti‐Cdk1 (#sc‐54, SantaCruz), goat anti‐Iba1 (#ab5076, Abcam), rabbit anti‐F4/80 (#GB113373, Servicebio), rabbit anti‐MPO (#ab208670, Abcam), rat anti‐CD44 (#550538, BD Biosciences), rabbit anti‐Dclk1 (#21699‐1‐AP, Proteintech). Sections were washed for three times with PBST (0.3% Triton X‐100 in PBS), and incubated in the appropriate secondary antibodies (Invitrogen) for 1 h at room temperature. The secondary antibodies used in the experiments included Alexa Fluor 488 Donkey anti‐Rabbit (#A21206), Alexa Fluor 594 Donkey anti‐Mouse (#A‐21203), Alexa Fluor 594 Donkey anti‐Goat (#A11058), Alexa Fluor 594 Donkey anti‐Rat (#A21209), Alexa Fluor 488 Donkey anti‐Mouse (#A21202), Alexa Fluor 568 Donkey anti‐rabbit (#A10042), and Alexa Fluor 647 Donkey anti‐rabbit IgG (#A31573).

For immunostaining of cultured organoids, they were collected through centrifugation, washed with PBS, fixed in 4% PFA for 20 min, and then dehydrated in 30% sucrose at 4°C overnight. The organoids were wrapped in warm gelatin/sucrose solution and equilibrated at 37°C for 15 min followed by solidification at −20°C. The solidified organoid‐gelatin/sucrose complex was embedded in O.C.T. compound, and 20‐μm‐thick sections were prepared using a Cryostat (model CM1950, Leica). Sections were washed with PBS, followed by incubation with blocking buffer containing SuperBlock (Thermo Fisher Scientific), 2% (v/v) donkey serum, and 0.3% Triton X‐100 at room temperature for 1 h. Primary antibody incubation was performed overnight at 4°C. After washing with PBS, appropriate secondary antibodies were used to visualise the staining. The primary antibodies used were as follows: rabbit anti‐OMP, mouse anti‐Tuj1, goat anti‐IL33, rabbit anti‐PGP9.5 (#14730–1‐AP, Proteintech), mouse anti‐Ki67, mouse anti‐Trp63 (#ab735, Abcam).

The cell nuclei were counterstained with DAPI (Thermo Fisher Scientific). Sections were mounted with Vectashield (Vector Laboratories, #H‐1000). Fluorescent images were captured using a confocal microscope (Model SP8, Leica) with LAS AF Lite software.

### 2.3 RNAscope

The tissue sections were washed in PBS to remove O.C.T., followed by dehydration in 50%, 70%, and 100% ethanol sequentially. Then, the sections were preprocessed with RNAscope hydrogen peroxide, target repair reagent, and Protease III to increase tissue permeability and promote the binding of RNAscope probes to target RNA in the OE. The RNAscope probe hybridization was performed at 40°C in the HybEZTN oven after these pretreatment steps. The Chil4‐C2 probe (Bio‐techne, #1248991‐C2) was diluted at a ratio of 1:50, along with the Alox15‐C1 probe (Bio‐techne, #539781) or the Cxcl5‐C1 probe (Bio‐techne, #467441). Four to six drops of probe mix were added to the sections and incubated for 2 h. Then, 4–6 drops of RNAscope multichannel second‐generation fluorescent AMP1, AMP2, and AMP3 were added to the sections sequentially and incubated for 30, 30, and 15 min, respectively. The fluorescent labelling of the Horseradish Peroxidase (HRP)‐C1 channel probe was visualised by incubation with HRP‐C1, Opal 570 fluorescent dye, and HRP blocker sequentially for 30, 30, and 15 min. The fluorescent labeling of the HRP‐C2 channel probe was then visualised by incubation with HRP‐C2, Opal 520 fluorescent dye, and HRP blocker. Sections were washed with fresh 1х RNAscope washing buffer twice between each reagent. Finally, nuclei were counterstained with DAPI (ThermoFisher Scientific) and the tissues were mounted with Vectashield (Vector Laboratories). Fluorescent images were captured using a confocal microscope (Leica SP8) with LAS AF Lite software.

### 2.4 Dexamethasone (Dex) Treatment

Dexamethasone (Sigma‐Aldrich, #50022) was dissolved in dimethyl sulfoxide (DMSO) as a stock solution, and the working solution was prepared by dilution with saline. The final concentration of dexamethasone was 1 mg/kg body weight in animals. One dose of dexamethasone or DMSO (as control) was intraperitoneally injected into Chil4^−/−^ mice per day, and animals were sacrificed after seven consecutive injections.

### 2.5 Western Blot

Proteins were extracted from OE tissues using 100 μL of RIPA (Radio Immunoprecipitation Assay) lysis buffer containing Phenylmethanesulfonyl fluoride (PMSF) protease inhibitor (Beyotime). Samples were ultrasonically cracked on ice before centrifugation at 12,000 *g* at 4°C for 15 min. The supernatant was collected and boiled in 5 × SDS loading buffer at 100°C for 10 min. Proteins (5 μg) were separated by 10% SDS‐PAGE, and then transferred to Polyvinylidene fluoride (PVDF) membranes (Millipore). After incubation with blocking solution (Beyotime) for 30 min, the membranes were incubated overnight with primary antibodies at 4°C, and then incubated at room temperature with horseradish peroxidase (HRP)‐conjugated appropriate secondary antibodies for 1 h. Primary antibodies used to detect protein expression were anti‐Chil4 (Abcam, #ab192029) and anti‐β‐actin (Sangon Biotech, #D191047). The immunoreactive protein bands were visualised using a chemiluminescent imaging system (Tanon, #4600SF).

### 2.6 Microelectrode Array Recordings and Signal Analysis

In vitro electrophysiological recordings of OE tissues were made using a microelectrode array (MEA) system (MEA2100, Multi Channel Systems). After incubation, the tissue was mounted onto a custom‐designed planar MEA (60 electrodes, 200 μm spacing, 30 μm diameter). In order to improve the contact of the tissue to the electrodes, the tissue was held in place with a slice anchor (ALA HSG‐5BD, glass‐coated steel ring with polymidde‐coated silica fibres, Multi Channel Systems). The tissue was allowed to settle on the MEA for at least 10 min before recording and continuously perfused with oxygenated Ringer's solution. During recordings, Ringer's solution was removed, and odour stimulus (70 μL) was applied to the tissue using a pipette. Baseline spontaneous activity or stimulus‐evoked activity in the OE was recorded for 3 min. After each 3 min recording, the tissue was rinsed at least three times with oxygenated Ringer's solution and allowed to recover for 3 min before continuing with additional stimuli. Odours were dissolved as 10 mM stock solution in water and DMSO. Odour Mix1 contained eugenol, geraniol, allyl phenylacetate, 1‐octanol, benzyl acetate, (R)‐(−)‐carvone, 2‐heptanone, and citral, while Mix2 included octanoic acid, heptanoic acid, coumarin, linalool, octanal, β‐citronellol, (S)‐(+)‐carvone, and trans‐cinnamaldehyde. In mammals, the OE is directly exposed to the external environment. Therefore, the temperature of the MEA culture chamber was maintained at 28°C using the integrated heating element of the MEA2100 amplifier. Signals were amplified (×1200), digitised (1 kHz), filtered (< 500 Hz), stored, and exported as axon binary files for offline analyses with custom‐written MATLAB (The MathWorks) scripts.

Raw signals were band‐filtered (0.1–300 Hz) using the zero‐phase digital filter ‘filtfilt’ to prevent phase distortion. In our previous studies, we demonstrated that odours mainly evoked slow oscillation (< 12 Hz) in the OE in vitro [27]. Therefore, we focused analysis on low‐frequency local field potentials (LFPs, 0.1–12 Hz) in this study. Every 3 min, the signal was cut into non‐overlapping 10‐s‐long segments. The band power of each segment was calculated using the ‘bandpower’ function. Results were then averaged across all segments. The recorded LFP response was converted to Δ*P*, where Δ*P* was calculated by subtracting the averaged LFP (in the 0.3–12 Hz range) power before odour presentation from the averaged power during odour presentation. The power change was proportional to the change in amplitude of slow oscillations.

### 2.7 OE Organoid Culture

OE tissues were dissected from Chil4^−/−^ (3‐month‐old), young WT (3‐month‐old) and aged WT (24‐month‐old) mice and digested in 0.25% trypsin–EDTA for 30 min at 37°C to prepare the single‐cell suspension. Cells were cultured in Matrigel (BD Biosciences) drops with the OE organoid growth medium containing DMEM/F12 medium (Gibco, #10565018) supplemented with R‐Spondin‐1 (200 ng/mL; R&D, #4645‐RS), Noggin (100 ng/mL; PeproTech, #250–38), Wnt3a (50 ng/mL; R&D Systems, #5036‐WN‐010), Y27632 (10 μM; Sigma‐Aldrich, #Y‐0503), human epidermal growth factor (50 ng/mL; ThermoFisher Scientific, #PHG0311), N2 (1%; ThermoFisher Scientific, #17502048), B27 (2%; ThermoFisher Scientific, #17504044), HEPES (10 mM; ThermoFisher Scientific, #15630080), Penicillin–Streptomycin (1%, ThermoFisher Scientific, #15140122) and Primocin (100 μg/mL, Invivogen, #ant‐pm‐05). The medium was replaced every 3 days, and Matrigel was changed every 7 days. For organoid passaging, Cell Recovery Solution (Corning, #354253) was used to collect organoids from Matrigel drops, and Organoid Dissociation Reagent (Precedo, #PRS‐ODR) was used to prepare the single‐cell suspension. Organoids were passaged every 7–10 days based on their growth condition.

### 2.8 OE Organoid Differentiation

To induce neuronal differentiation, the OE organoid growth medium was replaced with differentiation medium at day 10 post‐culture and maintained for an additional 10 days. The OE organoid differentiation medium was based on growth medium supplemented with LY411575 (5 μM, Sigma‐Aldrich, #SML0506), CHIR99021 (3 μM, Sigma‐Aldrich, #SML1046), and retinoic acid (5 ng/mL, Sigma‐Aldrich, #R2625). The medium was changed every 3 days.

### 2.9 Adeno‐Associated Virus Preparation and Infection

Adeno‐associated virus (AAV) used in this study was prepared by PackGene Biotechnology (Guangzhou, China). Plasmids expressing three different short hairpin RNA (shRNA) which targeted different regions of the Chil4 sequence were constructed. The knockdown efficiency was verified using the HEK293T cell line overexpressing the full length of Chil4, and the shRNA with the highest downregulation efficiency (5′‐GAATAGGTGACCCTACTGTTA‐3′) was used to prepare AAV‐shChil4. The target transgene plasmids containing full‐length Chil4 or shRNA (shChil4) tagged with monomeric cherry fluorescent protein (mCherry) or Enhanced Green Fluorescent Protein (EGFP) were cloned into the AAV plasmid containing the CAG promoter and the WPRE cassette flanked by AAVDJ inverted terminal repeats. Aged OE organoids were infected with AAVDJ‐shChil4‐EGFP to knock down Chil4 and label infected cells, with AAVDJ‐EGFP serving as a control. Young OE organoids were infected with AAVDJ‐Chil4‐P2A‐mCherry encoding the full length of Chil4 to overexpress Chil4 and label infected cells, with AAVDJ‐mCherry serving as a control. At the 8th day of organoid expansion, 1.5 × 10^10^ GC AAV was added to the culture medium to infect approximately 900 OE organoids and maintained for 48 h. Then, the virus‐containing medium was removed, and organoids were cultured for another 10 days in differentiation medium before collecting for further analysis.

### 2.10 Single‐Cell Suspension Preparation for Sequencing

Intact nasal epitheliums of WT and Chil4^−/−^ mice at 3–4 months of age as well as aged WT mice at 22–24 months of age were dissected. All samples (OE tissues from three young vs. three aged animals, and from two WT mice vs. two Chil4^−/−^ mice) were minced with scissors into 1 mm^2^ pieces in PBS on ice, transferred into 15‐mL centrifuge tubes, and digested with 0.2% collagenase II and 0.25% trypsin–EDTA at 37°C for 1 h. A single‐cell suspension was prepared by pipetting with a fire‐polished glass tube, and the suspension was passed through 70‐mm and 40‐mm strainers (BD Falcon). Single cells were collected by centrifugation at 1500 rpm for 5 min at 4°C before being washed twice in cold PBS. The cells were resuspended in PBS with 0.04% bovine serum albumin and used for 10× Genomics sequencing.

### 2.11 Single‐Cell RNA Sequencing

Single‐cell suspensions were counted using a Luna fluorometer (Logos Biosystems) by Trypan dye staining or using a Countstar Cell Analyser (Alit Biotech) by AO/PI staining, ensuring more than 90% viability. For each OE tissue, ~ 15,000 cells were loaded into a Chromium Single Cell 3′ Chip (10× Genomics) and processed following the manufacturer's instructions. Single‐cell complementary DNA (cDNA) libraries were prepared using the Chromium Next GEM Single Cell 3′ Reagent Kit v3.1 according to the manufacturer's instructions by OE Biotech Co. Ltd. (Shanghai, China) and Novogene Co. Ltd. (Shanghai, China). Samples were run using the 10× Genomics platform based on the manufacturer's protocol. All libraries were sequenced on the NovaSeq 6000 or NovaSeq X Plus Sequencing System (Illumina).

### 2.12 Quality Control and Data Processing

The CellRanger software (version 7.1.0) from 10× Genomics was used to generate raw feature‐barcode matrices aligned to the mm10 genome. The resulting feature‐barcode matrices were read into R (version 4.2.2), excluding any cell expressing fewer than 200 genes and any gene expressed in fewer than three cells. We used the Seurat R package (version 4.0.3) to remove cells with greater than 10% mitochondrial mapping. Similarly, cells with fewer than 200 or more than 6000 genes were filtered out, resulting in 19,610 cells in the WT group (8168 cells in WT1, 11,442 cells in WT2) and 16,141 cells in the Chil4^−/−^ group (8398 cells in Chil4^−/−^1, 7743 cells in Chil4^−/−^2). Integration of the datasets was performed using the ‘IntegrateData’ function.

### 2.13 Cell Type Identification

The cell type identification was described in our previous report with some modifications [21]. The integrated dataset was normalised using the ‘NormalizeData’ function. The top 2000 variable features were identified using the ‘FindVariableFeatures’ function with the ‘vst’ selection method. Total cell clustering was performed by ‘FindNeighbors’ and ‘FindClusters’ functions using the first 20 PCs (determined by the ‘Elbow plot’ and ‘DimHeatmap’ functions) and a resolution of 1. Then, we used the ‘RunHarmony’ function in harmony R package (version 1.2.0) to calculate the corrected coordinates. Dimensional reduction was performed with the ‘RunTSNE’ function, and the reduction parameter was set to ‘harmony.’

For each cell type, we used multiple cell type‐specific marker genes to determine its identity. The following cell types were annotated (selected markers were listed): B cell (expressing Cd79a, Cd79b, Cd19), Bowman's gland (BG; expressing Muc5b), fibroblast stromal cell (FSC; expressing Lum, Dcn, Mgp), immature OSN (iOSN, expressing Gap43, Gng8), mature OSN (mOSN; expressing Omp, Stoml3), microvillous cell (MV; expressing Sox9, Trpm5, Ascl3, Cftr); myeloid cell (expressing Cd14, Cd68), HBC (expressing Krt5, Krt14, Trp63), GBC (expressing NeuroD1, NeuroG1), pericyte (expressing Eng, Sox17), respiratory ciliated cell (RCC; expressing Foxj1, Cfap126), respiratory gland progenitor cell (RGP; expressing Sox9, Tubb4b), respiratory basal cell (ResC; expressing Tubb4b, Krt5, Krt19), sustentacular cell (SUS, expressing Cyp2g1, Ermn, Sult1c1, Ugt2a1), olfactory ensheathing glia (OEG; expressing Sox9, S100b), T cell (expressing Cd3d, Cd3e, Il17r); neutrophil (expressing S100a8, S100a9, Csf3r); microglia (MG)‐like cell (expressing Aif1, Trem2, Hexb); monocyte (Mono, expressing H2‐Ab1, Ccr2, S100a4); respiratory cell (ResC, expressing Krt19); vascular smooth muscle cells (VSM, expressing Tag1n, Myh11). Potential doublets were identified via the DoubletFinder R package (version 2.0.3) and removed. Then, we removed a cluster marked by odorant binding protein encoding genes (expressing Obp1a, Obp1b) and an undetermined cluster.

The final dataset identified 34,037 cells for bioinformatic analyses, including 18,735 cells in the WT group (7753 cells in WT1, 10,982 cells in WT2) and 15,302 cells in the Chil4^−/−^ group (7860 cells in Chil4^−/−^1, 7442 cells in Chil4^−/−^2). For the annotation of GBC subpopulations, we subsetted the cell cluster from the Seurat object using the ‘subset’ function. New analyses were performed using the ‘FindVariableFeatures’, ‘ScaleData’, ‘RunPCA’, and ‘RunUMAP’ functions. The following GBC subtypes were identified (markers were listed): GBC1 (expressing Sox2, Pax6, Hes1, Ascl1), GBC2 (expressing Neurod1, Neurog1, Lhx2).

### 2.14 Identification of Differentially Expressed Genes

To identify differentially expressed genes (DEGs) between Chil4^−/−^ and WT groups in each specific cell type, we used the ‘FindMarkers’ function in the Seurat R package (version 4.0.3) with the non‐parametric two‐sided Wilcoxon rank‐sum test. Genes with an average log_2_ (fold change) (avg_log_2_ FC) greater than 0.2 and an adjusted *p* value less than 0.05 were considered as upregulated genes. Genes with an avg_log_2_ FC less than −0.2 and an adjusted *p* value less than 0.05 were considered as downregulated genes. All DEGs for each cell type were visualised by the ggplot2 R package (version 3.5.1). The DEGs for each specific cell type between aged and young groups were identified using the same method.

### 2.15 Chil4 Correlation Analysis

To evaluate the correlation between the expression of Chil4 and other genes within the scRNA‐seq dataset, the RNA expression matrix was extracted from the Seurat object and subjected to log_2_ transformation (log_2_ (dat + 1)) to normalise the data for downstream analysis. A for‐loop was employed to calculate the Spearman correlation coefficient between Chil4 and each of the other genes in the RNA expression matrix. For each gene, the correlation coefficient and its corresponding *p*‐value were computed using the ‘cor.test’ function. Genes were ranked in descending order based on their correlation coefficient, and the top 500 genes with significant *p* values (*p* < 0.05) were selected for further analysis. Finally, a dot plot was generated using the Seurat package to visualise the expression patterns of Chil4 and the selected correlated genes.

### 2.16 Gene Ontology Analysis

Gene Ontology (GO) analysis was performed using the clusterProfiler R package (version 4.6.2) and visualised with the ggplot2 R package (version 3.5.1). The symbol ids were translated into entrez ids using the ‘bitr’ function. GO enrichment analysis for DEGs was performed using the ‘enrichGO’ function, and representative GO terms selected from the top 30 ranked ones were displayed. GO enrichment analysis for multiple cell types was performed using the ‘compareCluster’ function. The upregulated top ranked GO terms were selected from ≥ 6 cell subtypes, while downregulated GO terms were selected from ≥ 5 cell subtypes.

### 2.17 Identification of Cluster‐Specific Markers

The ‘FindAllMarkers’ function in the Seurat package was used to identify cluster‐specific markers. Only genes with an expression percentage greater than 50% in cells were considered as markers. Then, we selected markers with an avg_log_2_ FC value greater than 1 to generate a heatmap. The ‘AverageExpression’ function was used to calculate the expression levels for each subpopulation markers. The ‘pheatmap’ function in the pheatmap R package (version 1.0.12) was used to draw a heatmap of cluster‐specific markers, and the scale variable was set to ‘row’.

### 2.18 Cell Cycle Related Gene Analysis

By using the ‘CellCycleScoring’ function from the Seurat package (version 4.0.3), we calculated S and G2/M phase scores for each cell based on the expression of the S and G2/M phase marker genes [21]. The function directly classifies each cell into the G2M phase or the S phase based on these scores, and cells that do not express either set of markers are predicted to be in the G1 phase. This classification was a direct output of the ‘CellCycleScoring’ function. We then used the ‘table’ function to count the number of cells in each phase and calculated the proportions using the ‘prop.table’ function. Finally, the results were visualised using the ggplot2 package.

### 2.19 Gene Set Enrichment Analysis

The ‘AddModuleScore’ function in Seurat R package (Version 4.0.3) was used to calculate gene set scores for each single cell. The number of control genes was set to 100. The gene set scores of different cell types in WT and Chil4^−/−^ groups were visualised by ggplot2 R package. Boxplots were used to depict the distribution of the gene set scores across different cell types. The difference between WT and Chil4^−/−^ groups was determined with the non‐parametric two‐sided Wilcoxon rank‐sum test by the ‘stat_compare_means’ function implemented in the ggpubr R Package (version 0.6.0), and visualised in the boxplots with significance levels annotated. We used the ‘facet_grid’ function to create facets for different cell types to compare the differences and statistical significance between WT and Chil4^−/−^ groups in the boxplots. The inflammatory response gene set was referred to from MSigDB (Systematic name: MM4923).

### 2.20 Intercellular Communication Analysis

We used CellChat (Version 1.6.1) to analyse intercellular communication among GBCs, iOSNs, and mOSNs. CellChat objects were created based on the normalised expression count matrix of WT and Chil4^−/−^ groups, and then merged by ‘mergeCellChat’ function. ‘CellChatDB.mouse’ was used as the ligand‐receptor interaction database. Cell–cell communication analysis was then performed via the CellChat default setting. The interaction strength among different cell types was achieved by ‘netVisual_circle’ function. The information flow value was visualised by ‘rankNet’ function using ‘comparison’ mode. The ‘netVisual_bubble’ function was used to calculate the alteration in ligand‐receptor pairs between WT and Chil4^−/−^ groups.

### 2.21 NicheNet Analysis

NicheNet intercellular communication analysis was performed by nichenet R package (Version 1.1.1). The ‘weighted_networks’, ‘ligand_target_matrix’, and ‘lr_network’ databases were converted to mouse gene symbols from their human ortholog genes by ‘convert_human_to_mouse_symbols’ function. The receiver cell population was defined as microglia‐like cells, while the sender cell population was defined as HBC from the Chil4^−/−^ group. The ‘get_expressed_genes’ function was used to identify expressed genes in the sender population, and only those with percentage threshold (PCT) > 0.5 were considered as expressed genes. The upregulated DEGs (avg_log_2_ FC > 0.2 and *p*_val < 0.05) between Chil4^−/−^ and WT microglia were served as potential receptors and targets to receivers. Then, upstream regulatory ligands and downstream target genes in the sender and receiver cells were determined using the ‘predict_ligand_activities’ and ‘get_weighted_ligand_target_links’ functions with default settings. To identify ligand/receptor pairs, the interaction potential of ligands and receptors was ascertained using the ‘lr_network’ database assembled in NicheNet. GO analysis of target genes to Hbegf/CD44 pair was performed by clusterProfiler R package (version 4.6.2), and visualised with ggplot2 R package (version 3.5.1). Representative terms selected from the top 30 ranked GO terms (*P* adjust < 0.05) were displayed.

### 2.22 Trajectory Inference and Analysis

We used Slingshot (Version 2.6.0) to generate principal curves from GBC1 to iOSN. We re‐clustered the GBCs from the Seurat object and identified GBC1, GBC2, and iOSN clusters. Then, the GBC1, GBC2, and iOSN clusters were used to construct the differentiation trajectory by Slingshot. The GBC1 cluster was chosen as the root node, and the iOSN cluster was chosen as the endpoint. To visualise the differential expression of G2/M phase genes between Chil4^−/−^ and WT groups along the trajectory, we used the ‘plotSmoothers’ function in the tradeSeq package (Version 1.12.0).

We also used Monocle3 (Version 1.3.1) to infer the differentiation trajectory from GBC to mOSN. WT and Chil4^−/−^ cells (GBC1, GBC2, iOSN, mOSN) were subsetted from the Seurat object. Uniform manifold approximation and projection (UMAP) plots were generated for each subpopulation. No further batch correction or dimensionality reduction was performed in Monocle3. We specified the root of the trajectory by selecting the node most enriched for GBC1 cells. The trajectory and its direction calculated by Monocle3 were consistent with OSN development.

Trajectory inference was performed using velocyto.R (Version 0.6). The spliced and unspliced data were extracted from loom files constructed from raw data for each individual mouse OE. For calculating cell distance for k‐nearest neighbour (kNN) pooling, UMAP coordinates were extracted for WT and Chil4^−/−^ groups and passed to the ‘as.dist’ function in stats (Version 4.2.2). Then RNA velocities were estimated by ‘gene.relative.velocity.estimates’ function. To visualise RNA velocities on UMAP embedding, the ‘show.velocity.on.embedding.cor’ function was performed. The velocity scale was set to ‘linear’ mode for the GBC‐to‐mOSN trajectory analysis. For the GBC‐to‐iOSN trajectory analysis, the ‘sqrt’ mode was used.

### 2.23 Statistical Analysis

Three mice from different litters were used in each immunostaining and in situ hybridization experiment unless specified. Cells residing in the dorsal, lateral, and medial zones of both anterior and posterior OE were quantified. Generally, the number of positively stained cells per 100 μm OE, or OE plus lamina propria (LP), was quantified. Statistical analysis was performed by Microsoft Excel and GraphPad Prism 8.0.2 software. An unpaired *t*‐test was used to analyse the statistical significance, while the statistical significance in scRNA‐Seq data was determined by a non‐parametric two‐sided Wilcoxon rank‐sum test. All data were presented as mean ± standard error of the mean (SEM). *p* < 0.05 was considered statistically significant, and *p < 0.05, **p < 0.01, ***p < 0.001, ****p < 0.0001.

## 3 Results

### 3.1 Upregulation of Chil4 in the Aged OE

**FIGURE 1:**
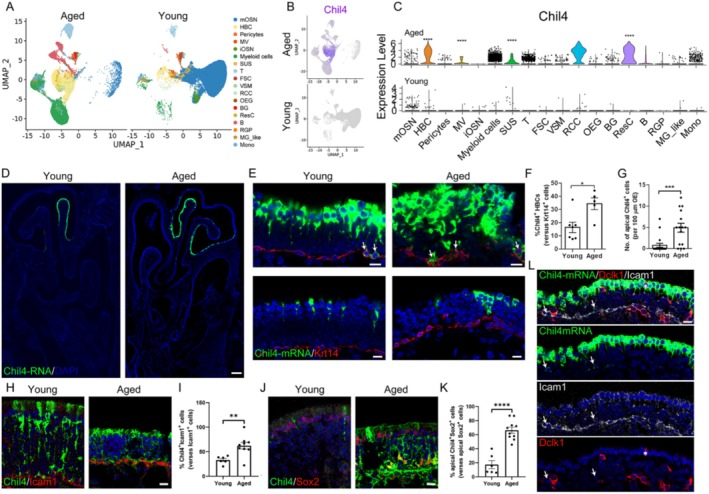
Upregulation of Chil4 in the aged OE. (A) UMAP plot showing cell subtypes in the aged and young OE. (B) UMAP plot showing Chil4 expression in the young and aged OE. (C) Violin plot showing Chil4 expression in different cell types of the young and aged OE. (D, E) Confocal images of Chil4‐mRNA^+^ and Krt14^+^ cells in the young (aged 3–4 months) and aged (aged 22–24 months) OE. (F, G) Quantification of basal (F) and apical (G) Chil4^+^ cells. *n* = 7 and 5 sections in the young and aged group in (F), *n* = 18 and 15 sections in (G). (H, J) Confocal images of Chil4^+^, Icam1^+^, Sox2^+^ cells in the young and aged OE. (I, K) Quantification of Chil4^+^Icam1^+^ cells and apical Chil4^+^Sox2^+^ cells. *n* = 6 and 9 sections in (I), *n* = 6 and 10 sections in (K). (L) Confocal images of Chil4^+^, Icam1^+^, and Dclk1^+^ cells in the OE. Arrows indicate Chil4^+^ Icam1^+^ Dclk1^−^ cells in the basal cell layer, while the asterisk notes Chil4^+^ Dclk1^+^ cell in the supporting cell layer. The statistical significance was determined by non‐parametric two‐sided Wilcoxon rank‐sum test in (C) and unpaired t test in (F, G, I, K). *p < 0.05, **p < 0.01, ***p < 0.001 and ****p < 0.0001. Scale bars: 200 μm in (D), 10 μm in (E, H, J, L).

To determine the role of Chil4 in aging, we performed scRNA‐Seq in the aged and young OE. UMAP plot showed that it captured main cell types constituting the OE (Figure 1A). Through scRNA‐Seq analysis, we found that Chil4 was upregulated in the aged OE compared to the young tissue (Figure 1B). The expression level of Chil4 was higher in horizontal basal cell (HBC), sustentacular cell (SUS), and respiratory ciliated cell (RCC) of the aged OE in contrast to young tissue (Figure 1C). The higher Chil4 expression was validated by RNAscope, showing that Chil4‐RNA expression was upregulated in the dorsal and dorsolateral zone of the anterior part, and mainly in the dorsolateral zone of the posterior OE, with aging (Figures 1D and S1). In the aged OE, Chil4 expression was obvious in the apical and basal cell layer, with a 5.3 ± 1.3‐fold (*p* = 0.0005) and 1.1 ± 0.3‐fold (*p* = 0.0134) increase in the number of Chil4‐RNA^+^ cells in the apical and Krt14^+^ HBC layer per 100 μm OE, compared to young tissue (Figure 1E–G). Immunostaining data also supported the increase in Chil4 expression at the protein level in the aged OE, showing an 88% ± 26% increase in the percentage of Chil4^+^Icam1^+^ HBCs (*p* = 0.0055, Figure 1H,I) and a 2.8 ± 0.4‐fold increase in Chil4^+^Sox2^+^ SUSs (*p* < 0.0001, Figure 1J,K). Chil4 was expressed in Icam1^+^ HBCs rather than the end feet of Dclk1^+^ microvillous cells in the OE (Figure 1L). Collectively, these data show aging‐related Chil4 upregulation, suggesting potential roles of Chil4 in OE aging and tissue homeostasis.

### 3.2 Upregulation of Chil4‐Related Genes Alox15 and Cxcl5 in the Aged OE

To further determine which gene was associated with high expression of Chil4 in the aged OE, we identified a list of Chil4‐related genes by performing Chil4 correlation analysis. Our scRNA‐Seq data analysis showed that these Chil4‐related genes in the aged OE were abundant in SUS, HBC, and RCC (Figure S2A). GO analysis demonstrated that Chil4‐related genes were enriched in the regulation of humoral response, inflammatory response, and epithelial cell differentiation (Figure S2B). KEGG analysis indicated that Chil4‐related genes were involved in Wnt, GTPase, and cytokine‐mediated signalling pathways in the aged but not young OE (Figure S2C). Compared to Chil4^−^ HBCs in the aged OE, Chil4^+^ HBCs showed higher expression levels of complement C3, Cxcl5, Alox15, and C‐C motif chemokine ligand 11 (Ccl11) (Figure S2D). This trend also occurred in Chil4^high^ HBCs of the aged OE, compared to Chil4^low^ HBCs (Figure S2E). Chil4^high^ and Chil4^low^ HBCs were defined as HBCs with Chil4 counts greater and less than the average value, respectively. Meanwhile, the expression level of Chil4‐related genes Cxcl5, Alox15, and C3 was significantly increased in aged HBCs compared to young cells (Figure S2F). Cytoscape showed Chil4 potentially interacted with Alox15 and other critical proteins involved in immunity and inflammation (Figure S2G). Additionally, higher expression levels of Alox15 in the aged HBCs and SUSs were confirmed by RNAscope and immunostaining data, showing an 88% ± 16% increase (*p* < 0.0001) in the number of Alox15^+^Krt14^+^ basal cells as well as a 43% ± 10% increase (*p* = 0.0024) in the number of Alox15^+^ cells in the apical layer (Figure S2H–K). The number of apical Chil4^+^Alox15^+^ cells in the aged OE was increased by 9.0 ± 2.3 folds (*p* = 0.0005) compared to the young tissue (Figure S2L). Besides, the number of Cxcl5^+^ cells was increased by 49% ± 10% (*p* < 0.0001) in the lamina propria (LP) of the aged mice compared to young ones (Figure S2I,M). Collectively, Chil4‐related genes such as Alox15 and Cxcl5 are upregulated in HBCs and apical cells of the aged OE, as well as in the LP.

### 3.3 Chil4 Deletion in the Young OE Impairs Tissue Homeostasis

**FIGURE 2:**
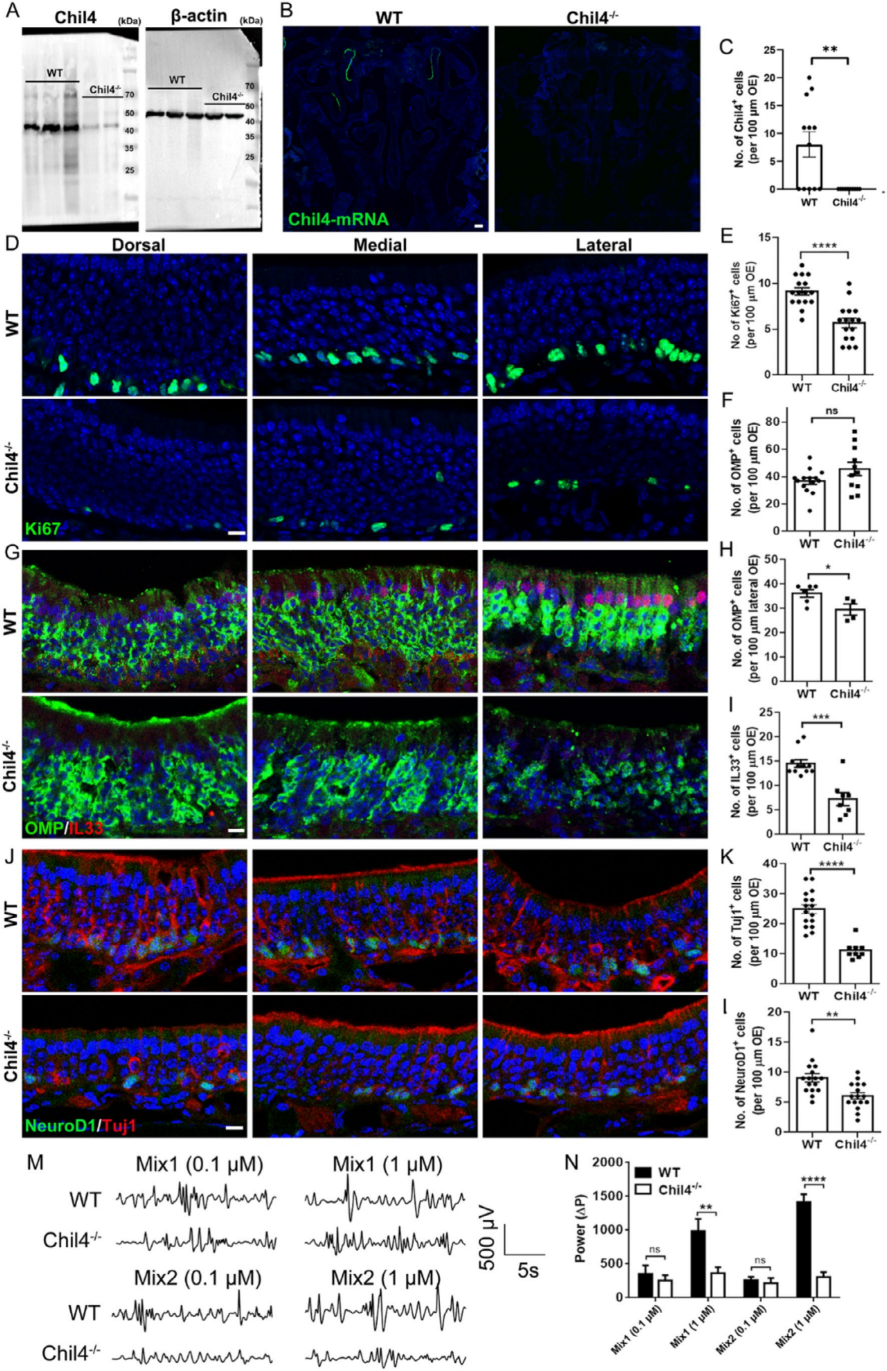
Chil4 knockout impairs olfactory epithelium homeostasis. (A) Western blot showing expression of Chil4 in the OE of WT and Chil4^−/−^ mice. (B, C) Confocal images and quantification of Chil4‐mRNA^+^ cells in the OE of WT and Chil4^−/−^ mice. *n* = 7 and 6 sections in WT and Chil4^−/−^ group. (D, E) Confocal images and quantification of Ki67^+^ cells in the dorsal, medial, and lateral OE of WT and Chil4^−/−^ mice. (G) Confocal images of IL33^+^, OMP^+^ cells in the dorsal, medial, and lateral OE of WT and Chil4^−/−^ mice. (F, H, I) Quantification of OMP^+^ OSNs in the OE (F) and in the lateral OE (H), IL33^+^ cells (I) in the OE of WT and Chil4^−/−^ mice. *n* = 14 and 11 sections in (F), *n* = 6 and 4 sections in (H), *n* = 11 and 8 sections in (I). (J) Confocal images of NeuroD1^+^ and Tuj1^+^ cells in the OE of WT and Chil4^−/−^ mice. (K, L) Quantification of Tuj1^+^ (K) and NeuroD1^+^ (L) cells in the OE of WT and Chil4^−/−^ mice. *n* = 16 and 9 sections in (K), *n* = 16 and 16 sections in (L). (M) The local field potential (LFP) of the OSNs to odour mixes in WT and Chil4^−/−^ mucosal tissues. (N) Quantification of the Δ*P* of LFP (Δ*P* = *P*_odor mix_ − *P*_baseline_). *n* = 8, 8, 7, 7 recordings for 0.1 μm Mix1, 1 μm Mix1, 0.1 μm Mix2, 1 μm Mix2 in WT group, *n* = 10, 10, 7, 7 recordings in Chil4^−/−^ group. The statistical significance was determined by unpaired t test. *p < 0.05, **p < 0.01, ***p < 0.001 and ****p < 0.0001. Scale bars: 200 μm in (B), 10 μm in (D, G, J).

The above scRNA‐Seq analysis and morphological experiments demonstrated that Chil4 and its related genes were upregulated with aging. However, the role of Chil4 in OE homeostasis remains unclear. In order to further determine how Chil4 functioned in OE homeostasis, we first investigated the changes in the cytoarchitecture of Chil4^−/−^ OE. Chil4 expression was significantly reduced in the OE of young Chil4^−/−^ mice compared to WT animals at both protein and mRNA levels (Figure 2A–C), providing evidence that Chil4 was completely knocked out in the Chil4^−/−^ OE. With Chil4 deletion, the number of Ki67^+^ proliferative cells was decreased by 36% ± 3% (*p* < 0.0001) throughout the OE compared to WT tissue (Figure 2D,E). The number of OMP^+^ mature sensory neurons showed a region‐specific change, with a reduction of 18% ± 6% (*p* = 0.0376) in the lateral region, but no significant change across the other regions (Figure 2F–H). The number of IL33^+^ SUSs was reduced by 50% ± 9% (*p* = 0.0001) in various OE regions, including dorsal, medial, and lateral parts (Figure 2G,I). Besides, the number of Tuj1^+^ immature neurons was reduced by 55% ± 4% (*p* < 0.0001) across various OE regions (Figure 2J,K). This was also supported by the reduction in the number of GAP43^+^ immature neurons by 38% ± 3% in the Chil4^−/−^ OE (Figure S3A,B, *p* < 0.0001). This decrease was potentially due to the reduction in the number of progenitor cells, since the number of NeuroD1^+^ GBCs across the OE was significantly decreased by 33% ± 8% with Chil4 deletion (Figure 2J,L, *p* = 0.0022). Furthermore, the number of HBCs was reduced by 15% ± 3% in the Chil4^−/−^ OE compared to WT tissue (*p* = 0.0035, Figure S3C,D), supporting our previous finding that Chil4 functioned in the OE regeneration [17].

We then asked whether Chil4 ablation impairs olfactory function by acutely isolating the OE and performing in vitro electrophysiological recordings. The responses to two odour mixes were quantified by the relative power (Δ*P*). Compared to WT controls, Chil4^−/−^ tissues showed significantly lower Δ*P* in response to the two odour mixes at 1 μM but not 0.1 μM (Figure 2M,N, *p* < 0.01 for Mix1, *p* < 0.0001 for Mix2), suggesting that Chil4 ablation reduces the functional response to odours in the OE. Moreover, Alox15 expression was significantly decreased in the apical layers of Chil4^−/−^ OE compared to WT tissue, with a 52% ± 6% (*p* = 0.0027) reduction in the number of Alox15^+^ cells in the apical cell layer (Figure S3E,F). Meanwhile, the number of Cxcl5^+^ cells was reduced by 48 ± 7% (*p* = 0.0012) in the LP of Chil4^−/−^ mice (Figure S3G,H). Thus, we conclude that the loss of Chil4 deteriorates OE tissue homeostasis primarily by targeting GBCs and immature sensory neurons, downregulates Chil4‐related genes, and impairs responses to odours.

### 3.4 Chil4 Regulates Growth and Neuronal Generation in OE Organoids

**FIGURE 3:**
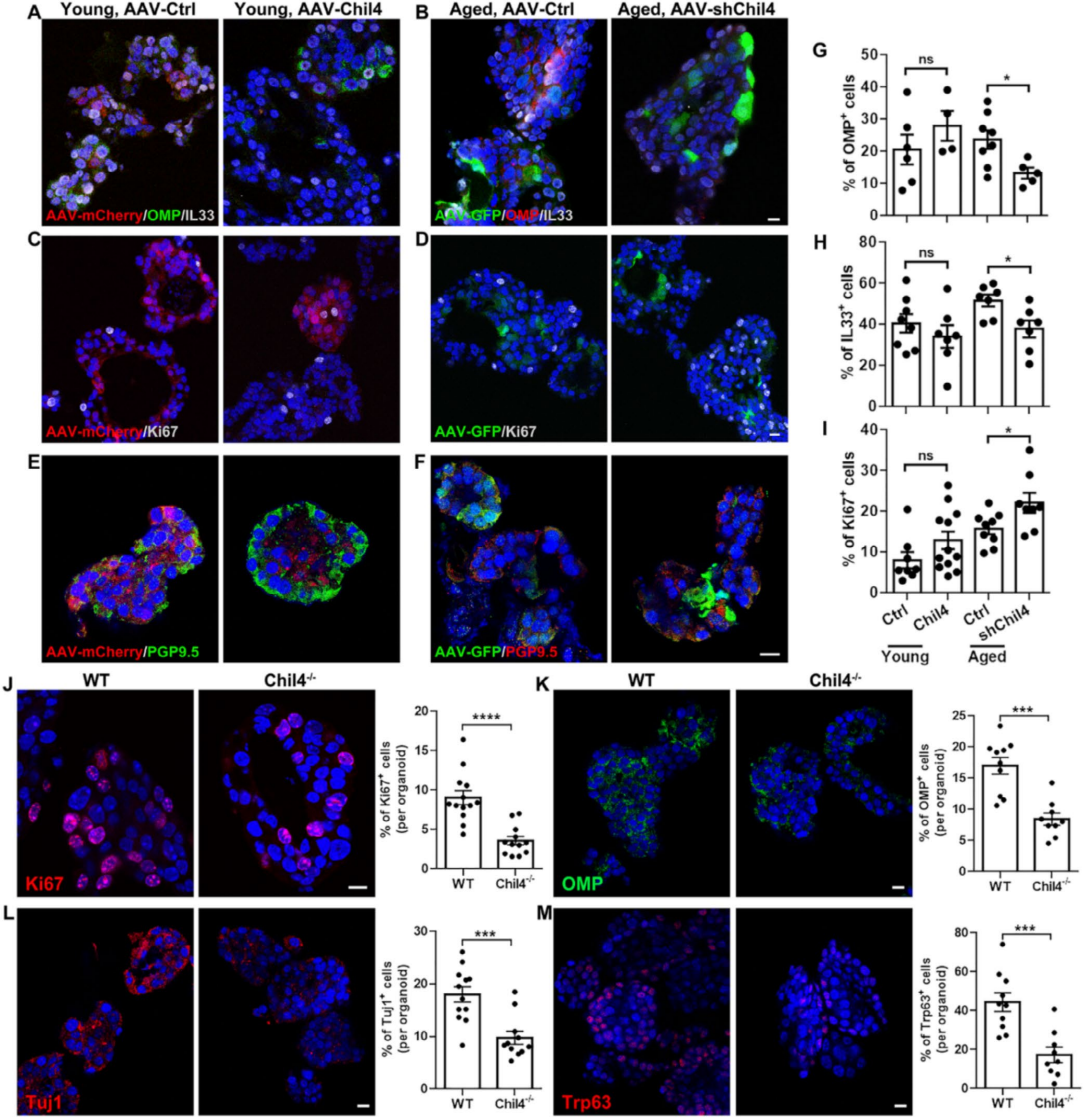
Chil4 regulates proliferation and neuronal generation in OE organoids. (A–F) Confocal images of OMP^+^, IL33^+^, Ki67^+^, PGP9.5^+^ cells in young organoids infected with control AAV‐mCherry or AAV‐Chil4‐mCherry (A, C, E), and in aged organoids infected with control AAV‐GFP or AAV‐shChli4‐GFP (B, D, F). (G–I) Quantification of OMP^+^ (G), IL33^+^ (H), Ki67^+^ (I) cells in young organoids infected with control AAV‐mCherry or AAV‐Chil4‐mCherry, and in aged organoids infected with control AAV‐GFP or AAV‐shChli4‐GFP. *n* = 6, 4, 8, 5 preparations in AAV‐Ctrl‐mCherry, AAV‐Chil4‐mCherry, AAV‐Ctrl‐GFP, AAV‐shChil4‐GFP group in (E), *n* = 8, 7, 7, 7 preparations in (F), *n* = 8, 12, 9, 8 preparations in (G). (J–M) Confocal images and quantification of Ki67^+^ (J), OMP^+^ (K), Tuj1^+^ (L), Trp63^+^ (M) cells in the WT and Chil4^−/−^ OE organoids. *n* = 13 and 12 preparations for WT and Chil4^−/−^ organoids in (J), *n* = 10 and 9 preparations in (K), *n* = 12 and 11 preparations in (L), *n* = 10 and 9 preparations in (M). Organoids in each group were derived from three animals. The statistical significance was determined by unpaired t test. ns, not significant, *p < 0.05, ***p < 0.001 and ****p < 0.0001. Scale bars: 10 μm.

The morphological and electrophysiological data in vivo described above suggest that Chil4 is necessary for young OE homeostasis. Then, we validated whether it plays the same role in OE organoids [28]. AAV expressing the full length of Chil4 (AAV‐Chil4) or shRNA targeting Chil4 (AAV‐shChil4) was used to regulate the Chil4 expression level in young and aged OE organoids. Overexpression of Chil4 in young organoids infected with AAV‐Chil4, as well as downregulation of Chil4 in aged organoids infected with AAV‐shChil4, were confirmed by RNAscope (Figure S4A,C). In aged organoids, Chil4 downregulation increased the organoid size by 23% ± 3% (*p* < 0.0001, Figure S4D). However, Chil4 overexpression in young organoids did not significantly change the organoid size (*p* = 0.4151, Figure S4B). These data suggest that Chil4 downregulation facilitated the growth of aged organoids, while overexpression of Chil4 did not affect the growth of young organoids. Chil4 downregulation in aged organoids significantly decreased the ratio of OMP^+^ cells by 44% ± 7% compared to the control group (*p* = 0.0218, Figure 3B,G), while overexpression of Chil4 in young organoids increased the ratio of OMP^+^ cells by ~35%, though this change was not significant (*p* = 0.3113, Figure 3A,G). However, no significant change was found in the number of PGP9.5^+^ (neuronal marker) cells in either viral‐infected aged or young organoids (Figure 3E,F). In addition, downregulation of Chil4 decreased the ratio of IL33^+^ cells by 27 ± 8% in aged organoids (*p* = 0.0175, Figure 3B,H), while Chil4 overexpression did not significantly change the ratio of IL33^+^ cells in young organoids (*p* = 0.3789, Figure 3A,H). Downregulation of Chil4 increased the ratio of Ki67^+^ proliferative cells by 41% ± 15% in aged organoids compared to the control group (*p* = 0.0328, Figure 3D,I). However, Chil4 overexpression did not significantly alter the ratio of Ki67^+^ cells in young organoids (*p* = 0.14, Figure 3C,I). Thus, we speculate that higher expression of Chil4 in aged organoids is essential for neuronal and sustentacular cell generation, but detrimental to cell proliferation.

To further elucidate the function of Chil4, we cultured OE organoids from WT and Chil4^−/−^ animals aged 3–4 months. The ratio of Ki67^+^ cells in Chil4^−/−^ organoids was reduced by 60% ± 6% compared to WT organoids, showing that Chil4 deletion impaired cell proliferation in young OE organoids (*p* < 0.0001, Figure 3J). Neuronal generation was attenuated in Chil4^−/−^ organoids, with a reduction of 50% ± 6% (*p* = 0.0001) and 46% ± 7% (*p* = 0.0004) in the ratio of OMP^+^ and Tuj1^+^ cells compared to WT organoids (Figure 3K,L). Additionally, the ratio of Trp63^+^ basal cells in Chil4^−/−^ organoids was decreased by 61% ± 9% compared to that in WT organoids (*p* = 0.0005, Figure 3M). Thus, Chil4 deletion attenuates neuronal generation and weakens stemness in OE organoids. These in vitro data are consistent with results from the Chil4^−/−^ OE.

### 3.5 Chil4 Deficiency Causes Gene Expression Changes in Multiple OE Cell Types

We then determined how Chil4 deficiency leads to transcriptional alteration in the OE at the single‐cell resolution by performing single‐cell RNA sequencing. The t‐SNE plot showed that the main cell types constituting the WT and Chil4^−/−^ OE were captured (Figure S5A). Dot plot visualisation of highly enriched genes in each cell subtype provided a panoramic view of annotated cell phenotypes (Figure S5B). We identified a subtype of cells expressing microglia‐specific markers, as well as common markers for microglia and macrophage. Since microglia is a cell type specific to the central nervous system, we termed these cells microglia (MG)‐like cells. In total, we identified 9840 differentially expressed genes (DEGs, |avg_log_2_FC| > 0.2 and *p*_val < 0.05) in various cell types between Chil4^−/−^ and WT OE. Some DEGs were globally changed across different cell subtypes, while others were cell‐type‐specific (Figure S5C,D). GBC, SUS, pericyte, MG‐like cell, and RCC in the Chil4^−/−^ OE possessed > 400 upregulated genes, while GBC, microvillous cell (MV), Bowman's gland cell (BG), and fibroblast/vascular smooth muscle cell (FB_VSM) had > 400 downregulated genes in the Chil4^−/−^ OE compared to WT tissue (Figure S5C,D). Thus, transcriptional alteration significantly appeared in these OE cell types with Chil4 deletion. To further delineate differential gene expression across OE cell types with Chil4 deletion, we identified 35 upregulated and 31 downregulated genes, and each was differentially expressed in at least 7 and 8 cell types of the Chil4^−/−^ OE compared to WT tissues, respectively (Figure S5E,F). The top 6 frequently upregulated genes included major urinary protein 4 (Mup4), odorant binding protein 1B (Obp1b), ribosomal protein S29 (Rps29), Obp1a, ribosomal protein L38 (Rpl38), and lipocalin 11 (Lcn11), while the top 6 shared downregulated genes were Obp2b, Nedd4 family interacting protein 1 (Ndfip1), S100 calcium binding protein A5 (S100a5), EP300 interacting inhibitor of differentiation 1 (Eid1), ADP‐ribosylation factor GTPase 5 (Arf5), and Mup5 (Figure S5E,F). Mup4, the most widely upregulated gene in the Chil4^−/−^ OE, functioned in pheromone binding [29]. Obp1a and Obp1b, significantly upregulated in Chil4^−/−^ iOSN and mOSN, were related to odorant binding (Figure S5G). S100a5, broadly downregulated by Chil4 deletion (Figure S5F,G), was an activity‐dependent gene in OSN [30]. Moreover, the downregulation of Eid1 in Chil4^−/−^ GBC potentially suggested impaired stem cell proliferation [31]. Ndfip1 is a neuroprotective protein [32], involved in the negative regulation of interleukin‐4 production [33], and limiting the production of proinflammatory cytokines [34]. Global downregulation of Ndfip1 in the Chil4^−/−^ OE may impair neuroprotective ability and lead to inflammatory activation. In addition, GO enrichment analysis revealed that upregulated genes in the Chil4^−/−^ OE were mainly related to ribosome biosynthesis and assembly, while downregulated genes were associated with the regulation of protein stability and protein folding (Figure S5H,I). Collectively, these data show that Chil4 deficiency leads to massive transcriptional change in the OE.

### 3.6 Chil4 Contributes to Cell Cycle Progression in GBC and Neuronal Differentiation Trajectory

**FIGURE 4:**
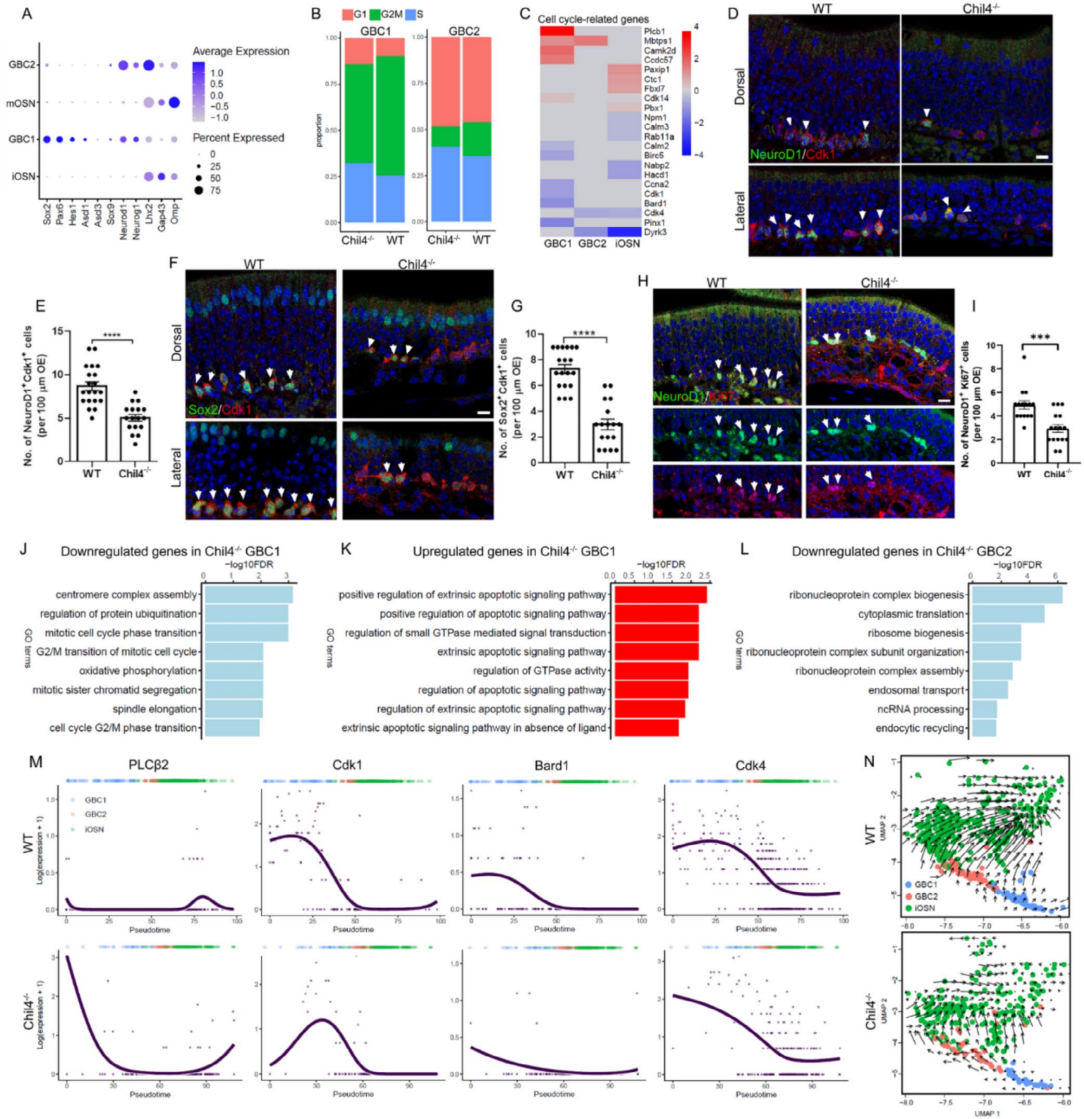
Chil4 deletion attenuates cell cycle progression in GBCs. (A) Dot plot showing the molecular markers for GBC1, GBC2, iOSN, and mOSN. (B) Proportions of GBC1 and GBC2 at G1, S, and G2/M phases. (C) Heatmap showing differential expression levels of cell cycle‐related genes in Chil4^−/−^ GBC1 and GBC2 compared to WT cells. (D, E) Confocal images (D) and quantification (E) of NeuroD1^+^ and Cdk1^+^ cells in the WT and Chil4^−/−^ OE. *n* = 20 and 17 sections in WT and Chil4^−/−^ sections. (F, G) Confocal images (F) and quantification (G) of Sox2^+^ and Cdk1^+^ cells in the WT and Chil4^−/−^ OE. *n* = 19 and 17 sections in WT and Chil4^−/−^ sections. (H, I) Confocal images (H) and quantification (I) of NeuroD1^+^ and Ki67^+^ cells in the WT and Chil4^−/−^ OE. *n* = 15 and 16 sections in WT and Chil4^−/−^ sections. (J–L) GO terms of downregulated (J) and upregulated (K) genes in Chil4^−/−^ GBC1, and of downregulated genes in Chil4^−/−^ GBC2 (L). (M) Slingshot showing the expression of cell cycle‐related genes along the trajectory from GBC to iOSN. (N) Stream plots of velocities on the UMAP of GBC and iOSN in the WT and Chil4^−/−^ OE. Arrows indicate differentiation directions and show the local average velocity. The statistical significance was determined by an unpaired *t* test. ***p < 0.001, ****p < 0.0001. Scale bars: 10 μm.

In the Chil4^−/−^ OE, the number of GBCs and iOSNs was significantly decreased (Figure 2). As iOSNs are derived from GBCs, we hypothesize that fewer GBCs in the Chil4^−/−^ OE enter the cell cycle, resulting in fewer generated iOSNs. We then performed cell cycle‐related gene analysis to confirm this hypothesis. Through scRNA‐Seq data analysis, GBCs were classified into two subtypes based on molecular markers (Figure 4A). The proportion of GBC1 in the G1 phase was much lower than that of GBC2, while the ratio of GBC1 in the G2/M phase was higher than GBC2 (Figure 4B). Compared to the WT group, the proportion of G1/S cells in both Chil4^−/−^ GBC1 and GBC2 was increased, while the proportion of G2/M cells was decreased (Figure 4B).

We then determined the differential expression of cell cycle‐related genes between Chil4^−/−^ and WT GBCs. Compared to WT cells, Chil4^−/−^ GBC1 displayed higher expression of calcium/calmodulin dependent protein kinase II delta (CAMk2d), a critical gene functioning in the G1/S transition of the mitotic cell cycle. Several genes important for cell cycle progression including baculoviral IAP repeat‐containing 5 (BirC5), cyclin dependent kinase 1 (Cdk1), and Cyclin A2 (Ccna2) were significantly downregulated in Chil4^−/−^ GBC1. Expression of BRCA1 associated RING domain 1(Bard1), which activates the G2/M checkpoint pathway, and calmodulin 2 (Calm2), which contributes to the G2/M transition of the mitotic cell cycle, was significantly reduced in GBC1 with Chil4 deficiency. By comparison, Chil4 knockout only downregulated the expression of Cdk4 and dual specificity tyrosine phosphorylation regulated kinase 3 (Dyrk3) in GBC2 compared to WT cells (Figure 4C). Immunostaining data further confirmed that the number of NeuroD1^+^Cdk1^+^ GBCs was significantly decreased by 39% ± 4% (*p* < 0.0001) in the Chil4^−/−^ OE compared to WT tissue (Figure 4D,E). Similarly, Chil4 deletion led to a reduction in the number of Sox2^+^Cdk1^+^ GBCs by 57% ± 2% (*p* < 0.0001) in the OE (Figure 4F,G). Chil4 deletion also reduced the number of NeuroD1^+^Ki67^+^ GBCs by 40% ± 10% (*p* = 0.0003) in the OE (Figure 4H,I). Additionally, GO analysis showed that downregulated genes in Chil4^−/−^ GBC1 compared to WT cells were involved in mitotic cell cycle regulation and protein ubiquitination, while upregulated genes functioned in the apoptotic signalling pathway (Figure 4J,K). By comparison, downregulated genes in Chil4^−/−^ GBC2 mainly participated in ribonucleoprotein biogenesis (Figure 4L). Several cell cycle‐related genes including phospholipase C beta 2 (PLCβ2), Cdk1, and Bard1 showed different expression patterns along the GBC differentiation trajectory between WT and Chil4^−/−^ OE (Figure 4M). Trajectory analysis using RNA velocity showed that GBC1 in the Chil4^−/−^ OE exhibited a weaker directional flow toward iOSN compared to WT GBC1 (Figure 4N).

**FIGURE 5:**
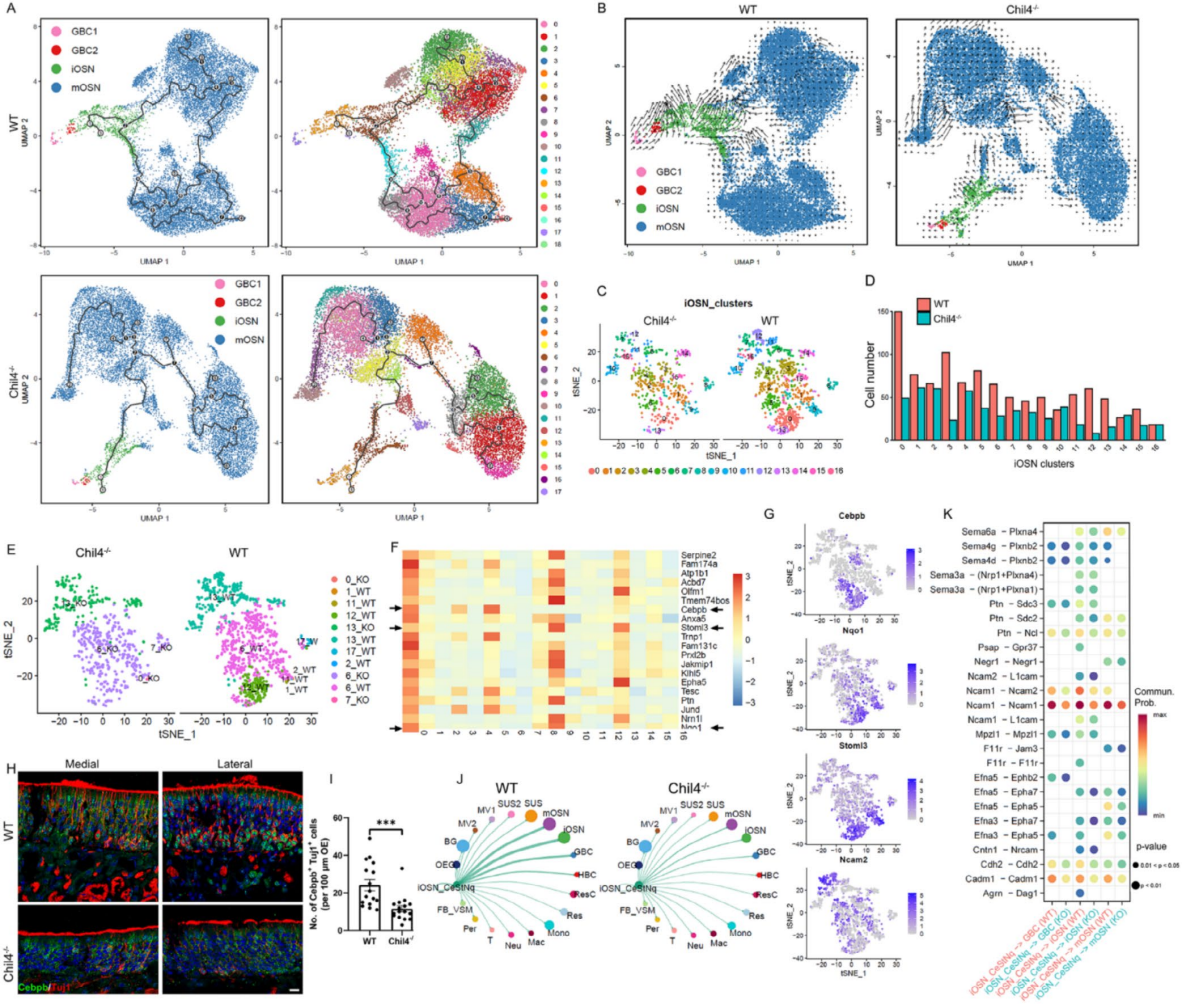
Chil4 deficiency causes loss of a unique type of iOSNs in the OE. (A) UMAP plots showing trajectory from GBC to mOSN in the WT and Chil4^−/−^ OE. (B) Stream plots of velocities on the UMAP of GBC, iOSN, and mOSN in the WT and Chil4^−/−^ OE. (C) Clustering of iOSNs in the WT and Chil4^−/−^ OE. The number of cells from subcluster‐0 to subcluster‐16 is 199, 137, 126, 125, 124, 118, 93, 84, 77, 75, 74, 71, 68, 63, 55, 53, and 36, respectively. (D) Cell number of different iOSN clusters in the WT and Chil4^−/−^ OE. (E) tSNE plot showing iOSN clusters using the same name as described in UMAP plot (A). (F) Heatmap showing highly expressed genes in each cluster of iOSNs. (G) tSNE plots showing expression of Cebpb, Stoml3, Nqo1, and Ncam2 in iOSN clusters. (H, I) Confocal images (H) and quantification (I) of Cebpb^+^ and Tuj1^+^ cells in the WT and Chil4^−/−^ OE. *n* = 16 and 16 sections in WT and Chil4^−/−^ sections. (J) Intercellular communication from iOSN expressing Cebpb, low level of Stoml3, and Nqo1 (iOSN_CeSt^L^Nq) to other cell types in the WT and Chil4^−/−^ OE by CellChat. (K) Decreased communication probability by ligand/receptor pairs from iOSN_CeSt^L^Nq to GBC, iOSN, and mOSN in the Chil4^−/−^ OE compared to WT tissue. The statistical significance was determined by unpaired t test. ***p < 0.001. Scale bars: 10 μm.

Moreover, pseudotime analysis showed a differential trajectory from GBC to mOSN with Chil4 deletion. A subcluster of iOSNs (cluster 12) in the WT OE was absent in the Chil4^−/−^ OE (Figure 5A). RNA velocity indicated that cluster 12 of iOSNs gave rise to mOSNs in the WT OE, and Chil4^−/−^ iOSNs showed a weaker flow toward mOSNs compared to WT iOSNs (Figure 5B). Clustering of iOSNs indicated that cell numbers of several subclusters were significantly decreased in the Chil4^−/−^ OE compared to the WT OE (Figure 5C,D). The cell cluster 12 from WT OE, which was absent in the Chil4^−/−^ OE, belonged to subcluster 0 of iOSNs (Figure 5A,C,E). This subcluster expressed a group of marker genes including CCAAT/enhancer binding protein beta (Cebpb, a transcriptional factor involved in inflammation and autophagy), NAD(P)H quinone dehydrogenase 1 (Nqo1, a marker for neurons located in the dorsal OE), and low level of Stomatin like 3 (Stoml3, a marker for mature OSN) (Figure 5F). This raised the possibility that this type of cell was transitioning to a mature state. However, subcluster 0‐iOSN did not highly express Neural cell adhesion molecule 2 (Ncam2), a marker for ventrally located neurons (Figure 5G). Immunostaining further confirmed that the number of Tuj1^+^ Cebpb^+^ neurons was decreased by 52% ± 14% in the Chil4^−/−^ OE compared to WT tissue (*p* < 0.0001, Figure 5H,I), confirming the loss of Cebpb^+^ iOSNs in the OE with Chil4 deletion. In addition, intercellular communication from subcluster 0‐iOSNs expressing Cebpb, low levels of Stoml3, and Nqo1 (iOSN_CeSt^L^Nq) to GBC, iOSN, or mOSN was apparently weaker in the Chil4^−/−^ OE compared to WT OE (Figure 5J). Cellchat analysis showed that communication from iOSN_CeSt^L^Nq to GBC, iOSN, or mOSN by ligand/receptor pairs related to neural activity was apparently weaker in the Chil4^−/−^ OE, including Sema6a/Plena4 for axonal guidance [35], Ncam1/Ncam1 for synapse maturation [36], and Efna/Epha for axon growth in OSNs [37] (Figure 5K).

Collectively, Chil4 deletion impairs cell cycle progression in GBC and changes the differentiation trajectory of iOSN, potentially leading to a deficit in neuronal homeostasis in the OE.

### 3.7 Chil4 Deficiency Promotes Inflammation in Microglia(MG)‐Like Cells

**FIGURE 6:**
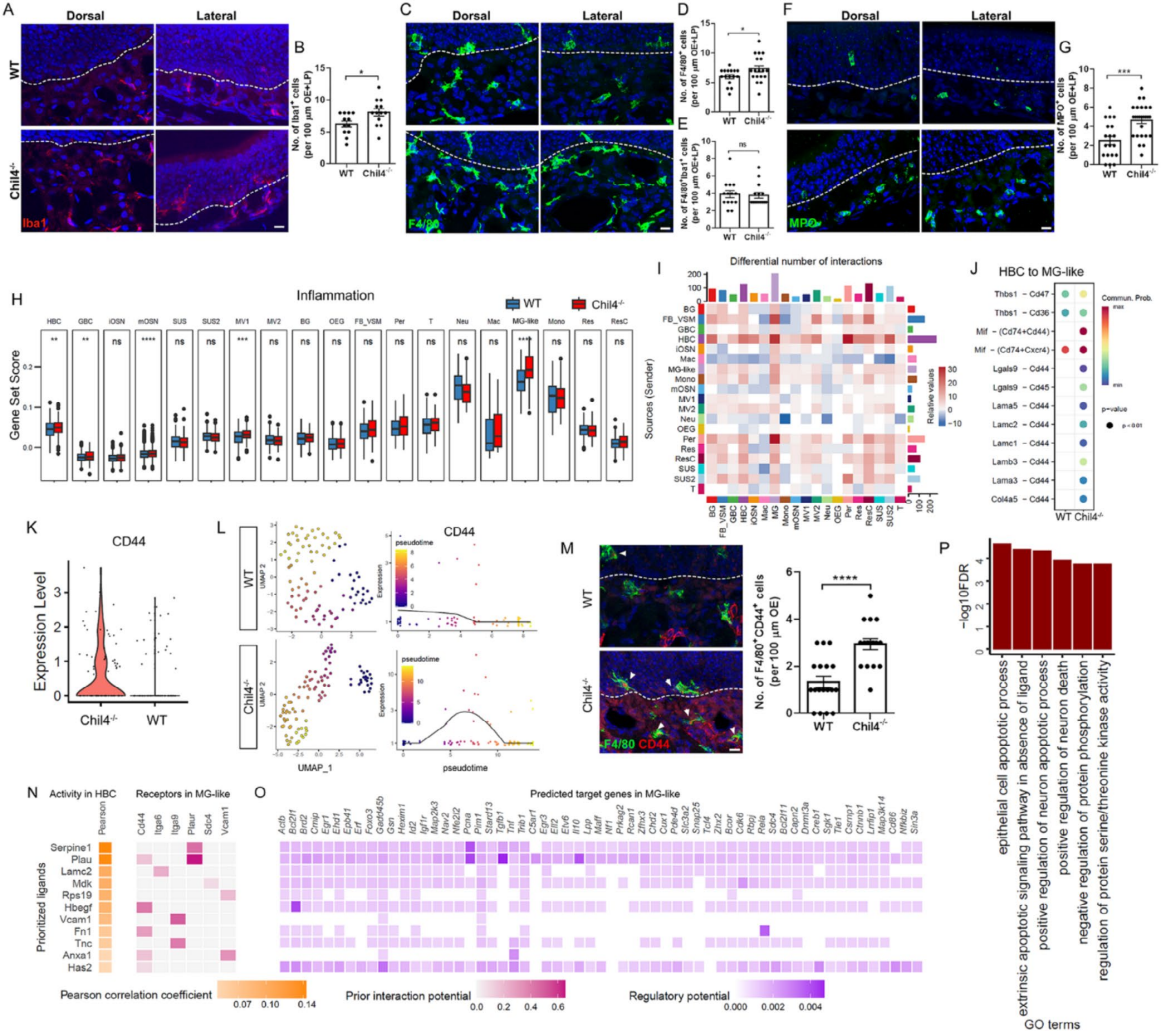
Chil4 deletion promotes inflammation in microglia. (A–G) Confocal images and quantification of Iba1^+^ (A, B), F4/80^+^ (C–E), and MPO^+^ (F, G) cells in the WT and Chil4^−/−^ OE. *n* = 12 and 13 sections for WT and Chil4^−/−^ groups in (B), *n* = 16 and 18 sections in (D), *n* = 14 and 15 sections in (E), *n* = 19 and 26 sections in (G). (H) Inflammation‐related gene set score in different cell types of WT and Chil4^−/−^ OE. (I) CellChat showing changes in the number of interactions between different OE cell types by Chil4 deletion. (J) Communication probability from HBC to microglia (MG)‐like cells through ligand/receptor pairs, predicted by CellChat. (K) Violin plot showing CD44 expression in WT and Chil4^−/−^ MG‐like cells. (L) Monocle3 showing CD44 expression along pseudotime in WT and Chil4^−/−^ MG‐like cells. (M) Confocal images and quantification of F4/80^+^ and CD44^+^ cells in the WT and Chil4^−/−^ OE. *n* = 18 and 17 sections for WT and Chil4^−/−^ groups. (N) Ligand activity in HBC and corresponding receptors upregulated in Chil4^−/−^ MG‐like cells, predicted by NicheNet. (O) Upregulated target genes to ligand/receptor pairs in Chil4^−/−^ MG‐like cells, predicted by NicheNet. (P) Go terms of upregulated target genes to Hbegf/CD44 pair in Chil4^−/−^ MG‐like cells. The statistical significance was determined by unpaired *t* test in (B, D, E, G, M), and by two‐sided nonparametric Wilcoxon's rank‐sum test with Bonferroni's correction in (H). ns, not significant, *p < 0.05, ***p < 0.001 and ****p < 0.0001. Scale bars: 10 μm.

Since our previous report showed that anti‐inflammation reduced Chil4 expression in the injured OE [17], we then determined whether Chil4 deficiency affected inflammation in the intact OE. In the Chil4^−/−^ OE, the number of Iba1^+^ MG‐like cells was increased by 29% ± 9% compared to that in the WT OE (*p* = 0.0249, Figure 6A,B). We also found that the number of F4/80^+^ macrophage/homeostatic microglia‐like cells was increased by 22% ± 8% in the Chil4^−/−^ OE (*p* = 0.048, Figure 6C,D). However, Chil4 deletion did not significantly change the number of Iba1 and F4/80 double‐positive cells (*p* = 0.8074, Figure 6E), potentially suggesting that Chil4 knockout preferentially affects active MG‐like cells in the OE. Besides, the number of MPO^+^ neutrophils was increased by 86% ± 13% in the Chil4^−/−^ OE compared to WT tissue (*p* = 0.0003, Figure 6F,G). The score of inflammation‐related gene set was significantly increased in MG‐like cells but not macrophages or neutrophils in the Chil4^−/−^ OE, suggesting a central role of MG‐like cells in inflammatory activation induced by Chil4 deletion (Figure 6H). Additionally, the intercellular communication from HBCs to MG‐like cells was apparently increased in the Chil4^−/−^ OE compared to WT tissue (Figure 6I). CellChat showed that communication between HBCs and MG‐like cells through the CD44 receptor expressed in MG‐like cells was enhanced in the Chil4^−/−^ OE (Figure 6J). Higher expression of CD44 was observed in Chil4^−/−^ MG‐like cells (Figure 6K), and pseudotime analysis by Monocle3 showed a dynamic change in CD44 expression along the MG‐like cell trajectory in the Chil4^−/−^ OE, but no fluctuation in CD44 expression level along the pseudotime trajectory in WT MG‐like cells (Figure 6L). Immunostaining further confirmed that the number of F4/80^+^ CD44^+^ cells was increased by 121% ± 17% in the Chil4^−/−^ OE compared to WT tissue (*p* < 0.0001, Figure 6M). NicheNet analysis indicated that heparin binding EGF like growth factor (Hbegf) expressed in HBCs bound to CD44 that was upregulated in Chil4^−/−^ MG‐like cells (Figure 6N). The target genes upregulated in Chil4^−/−^ MG‐like cells in response to the Hbegf/CD44 pair (Figure 6O) functioned in positive regulation of neuron apoptotic process and death (Figure 6P), suggesting that CD44 upregulation in Chil4^−/−^ MG‐like cells is likely to be associated with aberrant neuronal homeostasis in the OE.

Finally, to determine whether anti‐inflammation recovered OE homeostasis impaired by Chil4 deficiency, dexamethasone (Dex) or DMSO (as a control) was injected into Chil4^−/−^ mice. At Day 7 post injection, the number of Tuj1^+^ iOSNs across the Chil4^−/−^ OE was increased by 67% ± 12% in the Dex group compared to DMSO control (*p* = 0.0001, Figure S6A,B). Similarly, the number of GAP43^+^ immature neurons across the Chil4^−/−^ OE was increased by 36% ± 7% with Dex injection compared to controls receiving DMSO (*p* = 0.0023, Figure S6C,D). By contrast, the numbers of apical IL33^+^ and Sox2^+^ SUSs were not affected by Dex injection in the Chil4^−/−^ OE compared to DMSO control (Figure S6C,E). Moreover, the number of NeuroD1^+^ GBCs in the Chil4^−/−^ OE did not significantly change between Dex and DMSO groups (*p* = 0.0881, Figure S6E,F). Thus, we conclude that anti‐inflammation promotes the generation of immature neurons in the Chil4^−/−^ OE, while SUSs and GBCs are not recovered.

## 4 Discussion

**FIGURE 7:**
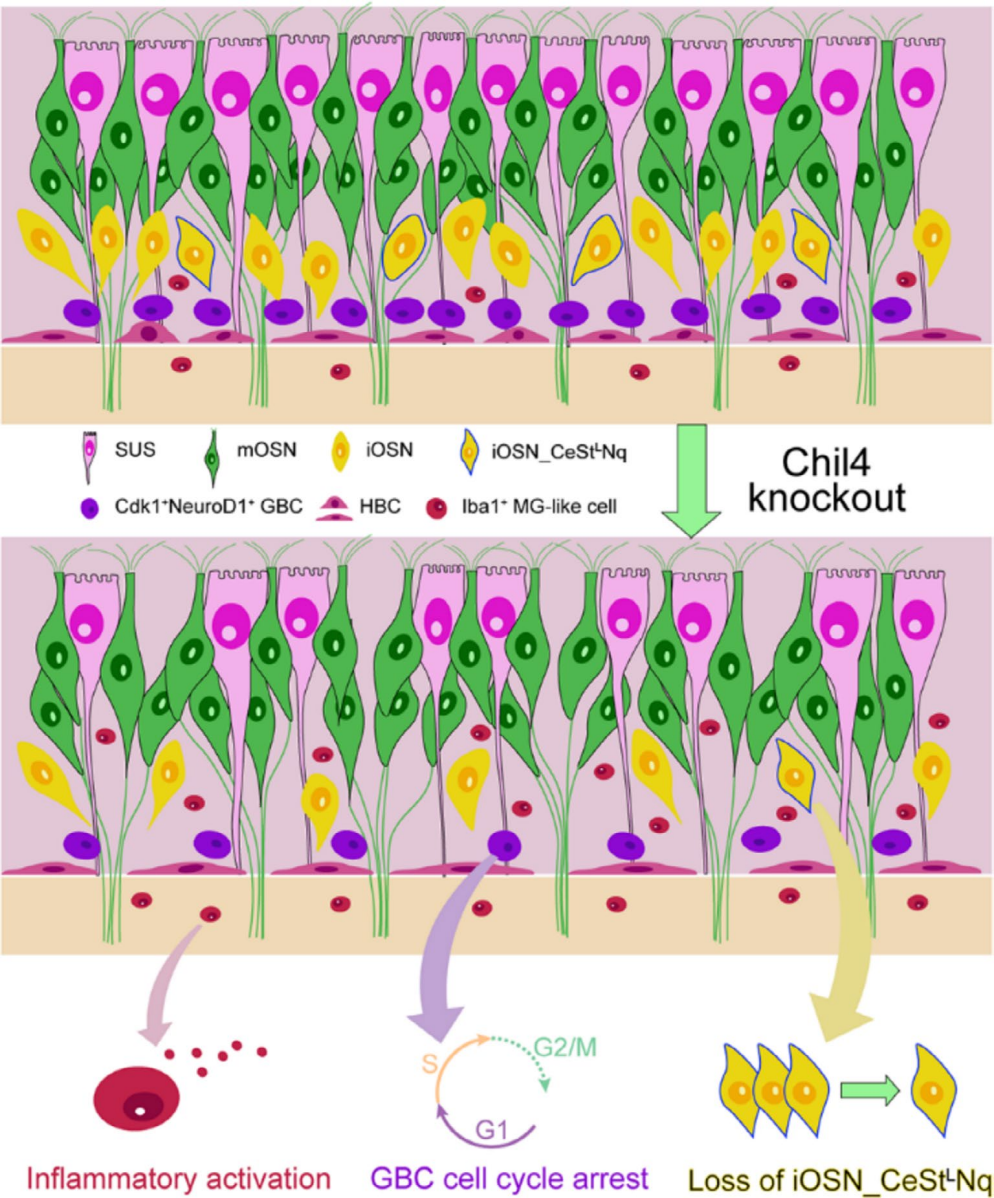
Summary of main findings in this study, showing the effect of Chil4 knockout on OE homeostasis.

Here, we described a previously unidentified role of Chil4 in OE homeostasis. Chil4 expression was elevated in the OE with aging. Chil4 deletion attenuated homeostasis of sensory neurons by reducing the number of GBCs and iOSNs. Chil4 deficiency in the OE attenuated cell cycle progression in GBCs, led to the loss of a unique cluster of iOSNs, and activated inflammation in MG‐like cells (summarised in Figure 7). We conclude that Chil4 is an important regulator of OE homeostasis.

Chil4, also known as Ch3il4 or Ym2, is a member of the glycoside hydrolase protein family that is specifically expressed in mice and plays a key role in the pathogenesis of certain inflammatory diseases. A previous study reported that Chil4 promoted Th2 cytokine production and caused allergic airway inflammation [11]. Chil4 was involved in regulating allergic diseases by responding to airway inflammation and hyperresponsiveness via Th2 inflammatory cells [38]. Elevated expression of chitinase‐like protein was observed post tissue injury [39, 40, 41], and overexpressed Chil4 was reported in airway injury and acute neutrophilic rhinitis models caused by ozone inhalation in rodents [42]. In addition, Choi et al. reported that reduced Chil4 expression in alveolar macrophages was helpful for suppressing the symptoms of asthma [43]. Besides, the repair process after tissue injury was often accompanied by acute inflammatory reactions and immune responses, while overexpression of chitinase‐like protein was reported to improve tissue repair [44]. Upregulation of Chil4 was reported in the early stage of Helicobacter‐induced gastric cancer, suggesting its role in tissue remodelling and wound healing [15, 45]. Our previous work showed that overexpression of Chil4 in the chemically lesioned OE accelerated the tissue recovery attenuated by anti‐inflammatory treatment [17]. Thus, Chil4 may play a bidirectional role in tissue regeneration and inflammatory responses post injury. Higher expression of Chil4 mainly in supporting cells post injury reported in our previous work suggested the potential cell type‐specific importance of this gene. By conditional knockout of Chil4 in supporting cells, we found that Chil4 was necessary for promoting OE regeneration via its interaction with inflammatory responses. In the current study, we found the complete knockout of Chil4 caused decreases in the numbers of several cell types, suggesting its necessity in OE homeostasis. We speculate that conditional knockout of Chil4 in supporting cells causes a deterioration in OE homeostasis, while further experiments will be performed in future studies.

In the aged OE, Chil4 was upregulated in SUSs and HBCs. There are two scenarios to explain the higher expression level of Chil4 with aging. Firstly, higher expression of Chil4 ensures normal olfactory function in aged animals. Secondly, higher expression of Chil4 in the aged OE leads to deterioration in olfaction. Based on the data presented in the current study, the first scenario has priority to explain the elevated expression of Chil4 in the aged OE. Using in vitro cultured OE organoids, we found that downregulation of Chil4 in aged organoids impaired neuronal and sustentacular cell generation, while overexpression of Chil4 in young organoids did not hinder cell differentiation and proliferation (Figure 3). These data suggest that higher expression of Chil4 is required for the homeostasis of aged OE. We inferred that high expression of Chil4 functions in maintaining homeostasis of the aged OE. However, the underlying mechanism needs more investigation, especially regarding how Chil4 impacts the death‐regeneration balance of neurons and inflammatory infiltration. This needs to be validated by studying the OE homeostasis in the aged Chil4^−/−^ mice in future studies.

Chil4‐related genes, such as Alox15, Cxcl5, C3, and Ccl11, were highly expressed in Chil4^+^ HBCs or HBCs with high Chil4 expression levels (Figure S2). In the Chil4^−/−^ OE, Alox15 and Cxcl5 expression was significantly downregulated compared to WT OE (Figure S3). Alox15^+^ macrophages contributed to type 2 immunity in eosinophilic chronic rhinosinusitis with nasal polyps, while inhibition of Alox15 alleviated type 2 inflammation [22]. The correlation between Alox15 and inflammation‐related genes was also established in chronic rhinosinusitis with nasal polyps [46]. Deletion of Alox15 could counteract tissue dysfunction [47]. Thus, we hypothesize that Alox15 downregulation is a compensatory response to Chil4 deletion to balance inflammation and tissue damage. Moreover, Cxcl5 served as an activator to drive inflammation [23], while suppression of Cxcl5 accelerated wound healing [48]. In the Chil4^−/−^ OE, downregulation of Cxcl5 may be a driving force behind the attenuation in cell generation. However, the modulatory correlation between Chil4 and its related genes needs to be confirmed by loss‐ or gain‐of‐function studies.

GBC play a central role in OE homeostasis, since new sensory neurons are generated from GBCs to replace old neurons. We found that Chil4 deletion led to a reduction in the number of GBCs and iOSNs across the OE (Figure 2). Cell cycle progression in GBCs was impaired by Chil4 deletion, with a reduction in the number of cells at the G2/M phase, and altered expression patterns of cell cycle‐related genes (Figure 4). Cdk1 plays a central role in cell cycle progression, driving cells through the G2 phase to mitosis [49]. Reduced number of Cdk1^+^ GBCs in the Chil4^−/−^ OE suggests fewer GBCs entering the M phase, potentially leading to fewer iOSNs generated from GBCs. Pseudotime analysis showed a higher level of Cdk1 in GBC1 but a gradual decrease in GBC2 and iOSNs of the WT OE, while Cdk1 expression in Chil4^−/−^ GBC1 fluctuated from low to high and then to low levels. This suggests that the aberrant expression pattern of Cdk1 in GBC1 is responsible for abnormal cell cycle progression. Further studies on regulating the cell cycle by Cdk1 upregulation in the Chil4^−/−^ OE will facilitate understanding how alteration in cell cycle progression affects the deficit in neuronal homeostasis induced by Chil4 deletion.

Acute and chronic inflammation exert differential effects on OE homeostasis and regeneration. For instance, long‐term stimulation by TNFα impaired the electrophysiological properties of olfactory sensory neurons [50], while a short period of increase in TNFα concentration was necessary for OE regeneration [19]. In this study, we found that the number of Iba1^+^ MG‐like cells was increased in the Chil4^−/−^ OE compared to WT tissue (Figure 6). Iba1^+^ microglia were activated in the olfactory bulb (OB) of an allergic rhinitis mouse model [51] and in the OB of mice exposed to toxic volatile organic compounds [52], suggesting that microglia in the olfactory tissue respond to lesions and inflammation. The role of microglia in inflammation was further supported by our finding that the inflammation‐related gene set score was increased in MG‐like cells of the Chil4^−/−^ OE (Figure 6). Combined with our previous data showing that Iba1^+^ MG‐like cells were activated in the Tmem59^−/−^ OE with an inflammatory environment [18], we conclude that the MG‐like cell is one of the major cell types mediating inflammation in the OE. Furthermore, we identified CD44 as an important receptor regulating intercellular communication from HBCs to MG‐like cells, and confirmed its higher expression level in Chil4^−/−^ cells by scRNA‐seq analysis and immunostaining (Figure 6). CD44 was reported to be a critical factor in inflammatory processes during wound healing [25], acute asthma [26], and atherosclerosis [53]. Elevated CD44 expression in MG‐like cells by Chil4 deletion may correlate with inflammatory activation and neuronal degeneration, thereby potentially contributing to the loss of iOSNs and impaired GBC cell cycle progression. Cebpb is a transcription factor (TF) regulating inflammation and autophagy in macrophages, and acts as a pleiotropic transcription activator of genes involved in energy metabolism, cell differentiation, and inflammation [54, 55]. Previous studies reported that Cebpb overexpression increased glycolysis and mitochondrial respiration in sensory neurons in disease‐associated rodent models [56], and neuroinflammation was suppressed by degrading Cebpb in microglia via the ubiquitin ligase COP1 [57]. We speculate that Cebpb downregulation in MG‐like cells is a compromising response to the activated inflammatory microenvironment under Chil4 ablation, aiming to maintain OE homeostasis as far as possible. However, more experiments are needed to uncover the mechanism in future studies. Further study targeting CD44 expression in the Chil4^−/−^ OE will provide direct evidence for its role in inflammation and neuronal homeostasis. Here, we used dexamethasone, a glucocorticoid medication used for anti‐inflammatory purposes in chronic rhinosinusitis [58], to block inflammatory activation. Anti‐inflammation improved iOSN generation in the Chil4^−/−^ OE, further supporting the notion that neuronal loss caused by Chil4 deletion is associated with inflammatory activation.

To confirm the role of Chil4 in OE homeostasis and aging, we cultured Chil4^−/−^ OE organoids and conducted AAV infection in young and aged organoids. The data presented in WT and Chil4^−/−^ organoids were consistent with those obtained from OE tissues, indicating that our conclusion regarding the function of Chil4 in the OE is reliable. Although the organoid model in vitro mimics tissue in vivo, it still shows a difference from the real organ. The lack of an inflammatory microenvironment in the OE organoid is the major discrepancy to epithelial tissue. Thus, the current in vitro model is not suitable for studying the role of Chil4 in inflammatory activation. Co‐culture systems that incorporate microglia into organoids have been established [59, 60, 61, 62], providing an inflammatory environment in organoids to facilitate studying the modulatory effect of inflammatory activation on tissue homeostasis and regeneration. We therefore will continue to improve the in vitro‐cultured OE organoid system by incorporating inflammatory cells to study the role of Chil4 in inflammatory activation and OE homeostasis.

## 5 Conclusion

Chil4 is required for OE homeostasis. Deficiency of Chil4 impairs OE homeostasis through loss of sustentacular cells and a specific subtype of immature sensory neurons, cell cycle arrest in globose basal cells and inflammatory activation in microglia‐like cells.

## Author Contributions

**Tingting Wu:** methodology, software, data curation, investigation, formal analysis. **Weihao Li:** methodology, software, investigation, data curation, formal analysis. **Liujing Zhuang:** data curation, formal analysis. **Jinxia Liu:** resources. **Ping Wang:** methodology. **Ye Gu:** conceptualisation. **Yongliang Liu:** conceptualisation, funding acquisition. **Yiqun Yu:** conceptualisation, resources, formal analysis, visualisation, supervision, writing – original draft preparation, writing – reviewing and editing, project administration, funding acquisition.

## Ethics Statement

The procedures of animal breeding and tissue harvesting were approved by the Committee of Laboratory Animals at Fudan University (Permit number: SYXK2020‐0032).

## Conflicts of Interest

The authors declare no conflicts of interest.

## Supporting information


**Data S1.** Supporting Information.

## Data Availability

The datasets generated and/or analysed during the current study are available in the China National GeneBank DataBase (CNGBdb) repository. The deposit number was CNP0005898. Other datasets used and/or analysed during the current study are available from the corresponding author (Y. Yu) on reasonable request.
