# Supplementary material for: Deficiency of Aging‐Related Gene Chitinase‐Like 4 Impairs Olfactory Epithelium Homeostasis
Source: Cell Prolif. 2025 May 19;58(8):e70055. doi: 10.1111/cpr.70055 (PMC12336453; doi:10.1111/cpr.70055)
Supplement: Supplementary file 1 — Data S1. Supporting Information. [file CPR-58-e70055-s001.pdf]

Supplementary figures for

Deficiency of aging-related gene Chitinase-like 4 impairs olfactory epithelium homeostasis

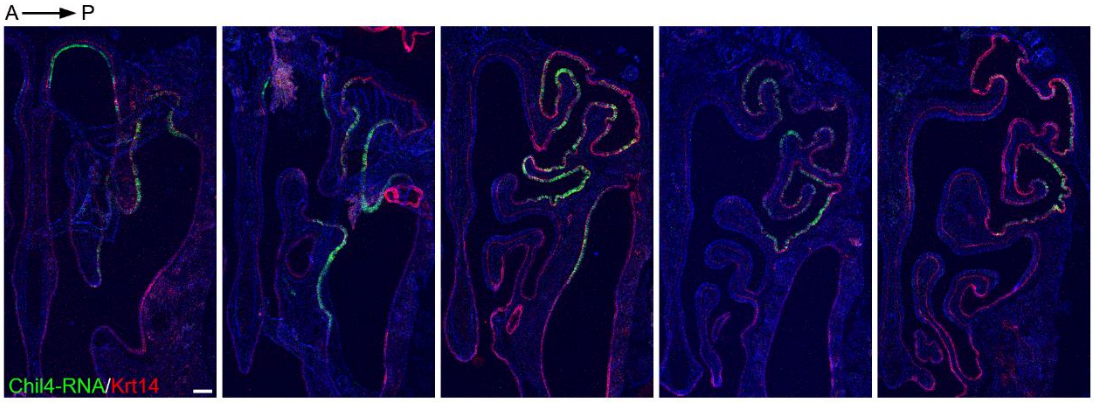

Figure S1. Confocal images of Chil4-RNA$^+$ cells in the aged OE from anterior (A) to posterior (P) region. Scale bar: 200 μm.

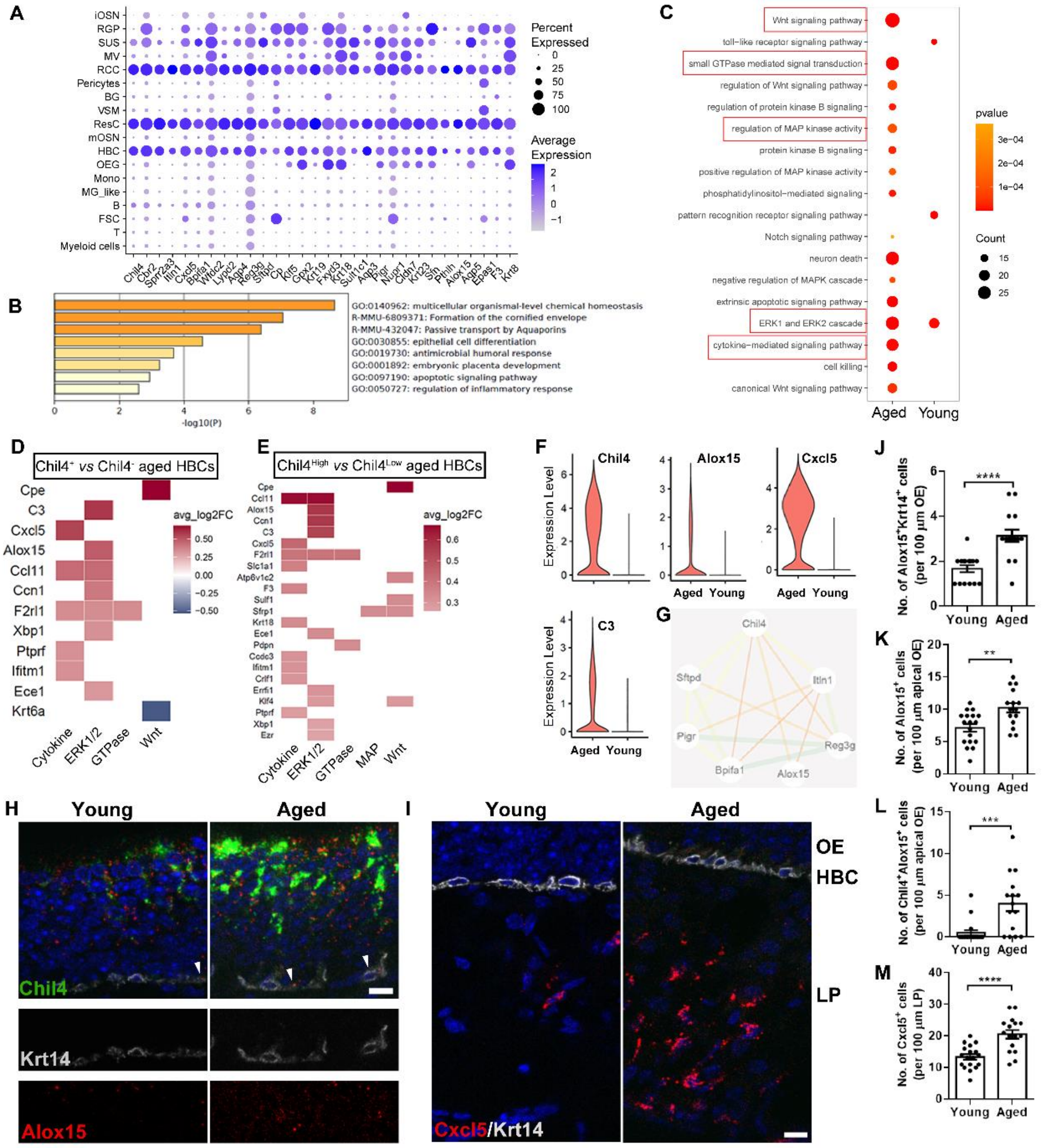

Figure S2. Elevated expression of Chil4-related genes in the aged OE. (A) Dot plot showing expression of Chil4-related genes in different cell types of the aged OE. (B) GO enrichment analysis of Chil4-related genes. (C) KEGG analysis of signaling pathways involved by Chil4-related genes in the young and aged OE. (D, E) Heatmap showing DEGs in cytokine, ERK, GTPase, MAP, Wnt signaling pathways between Chil4^{+} and Chil4^{-} (D), or Chil4^{High} and Chil4^{Low} (E) aged HBCs. (F) Violin plot showing expression of Chil4, Alox15, Cxcl5 and C3 in young and aged HBCs. (G) Cytoscape predicted interaction network among Chil4 and other genes. (H, I) Confocal images of Chil4-RNA^{+}, Krt14^{+}, Alox15-RNA^{+} (H), Cxcl5-RNA^{+} (I) cells in the young and aged OE. (J-M) Quantification of Alox15^{+}Krt14^{+} (J), apical Alox15^{+} (K), apical Chil4^{+}Alox15^{+} (L), Cxcl5^{+} cells (M). n = 15 and 15 sections in young and aged group in (J), n = 18 and 15 sections in (K), (L), n = 18 and 16 sections in (M). The statistical significance was determined by unpaired t test. **p < 0.01, ***p < 0.001, ****p < 0.0001. Scale bars: 10 μm.

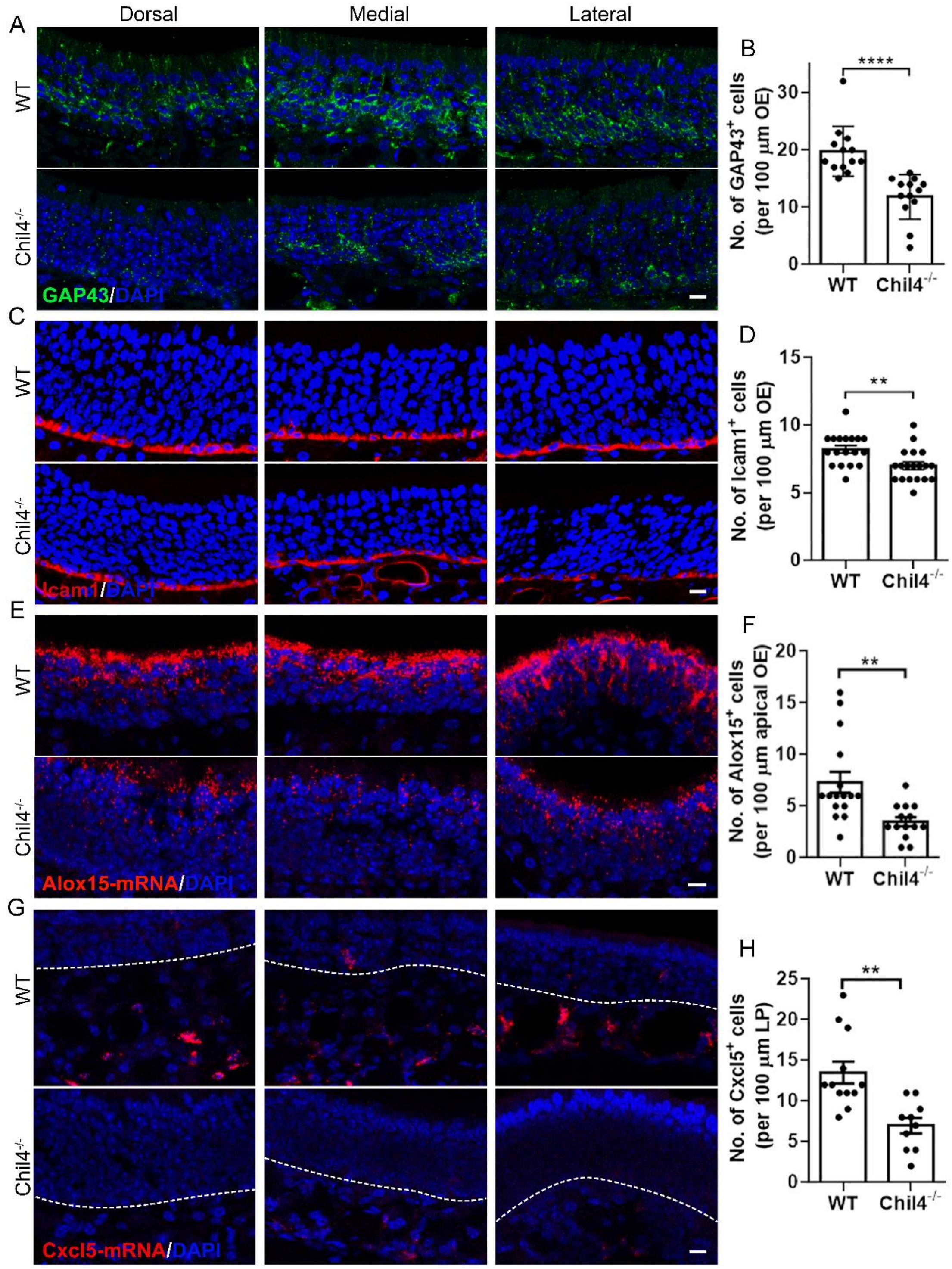

Figure S3. Chil4 deletion impairs expression of Chil4-related genes in the OE. (A, C, E, G) Confocal images of GAP43+ (A), Icam1$^+$ (C), Alox15-RNA$^+$ (E), Cxcl5-RNA$^+$ (G) cells in the WT and Chil4$^{-/-}$ OE. (B, D, F, H) Quantification of GAP43+ (B), Icam1$^+$ (D), Alox15-RNA$^+$ (F), Cxcl5-RNA$^+$ (H) cells in the WT and Chil4$^{-/-}$ OE. n = 13 and 13 sections for WT and Chil4$^{-/-}$ group in (B), n = 18 and 19 sections in (D), n = 16 and 14 sections in (F), n = 12 and 10 sections in (H). The statistical significance was determined by unpaired t test. **$p < 0.01$ and ****$p < 0.0001$. Scale bars: 10 μm.

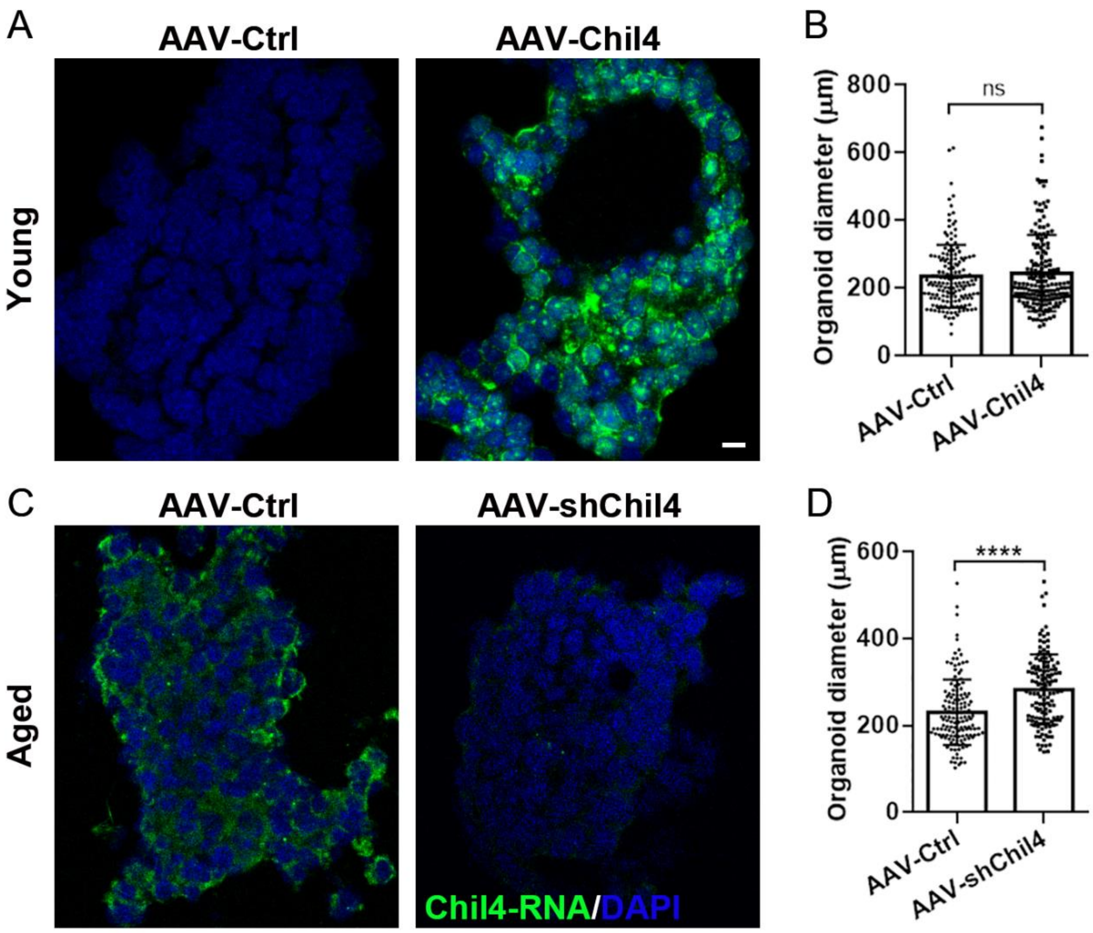

Figure S4. Chil4 regulates organoid growth. (A, C) Confocal images of Chil4^{+} cells in young organoids infected with AAV-Ctrl or AAV-Chil4 (A), and in aged organoids infected with AAV-Ctrl or AAV-shChil4 (C). (B, D) Quantitative analysis on diameter of young organoids infected with AAV-Ctrl or AAV-Chil4 (B), and of aged organoids infected with AAV-Ctrl or AAV-shChil4 (D). n = 155 and 172 organoids in AAV-Ctrl and AAV-Chil4 group in (B), n = 144 and 131 organoids in AAV-Ctrl and AAV-shCtrl group in (D). The statistical significance was determined by unpaired t test. ns, not significant, ****$p < 0.0001$, Scale bar: 10 μm.

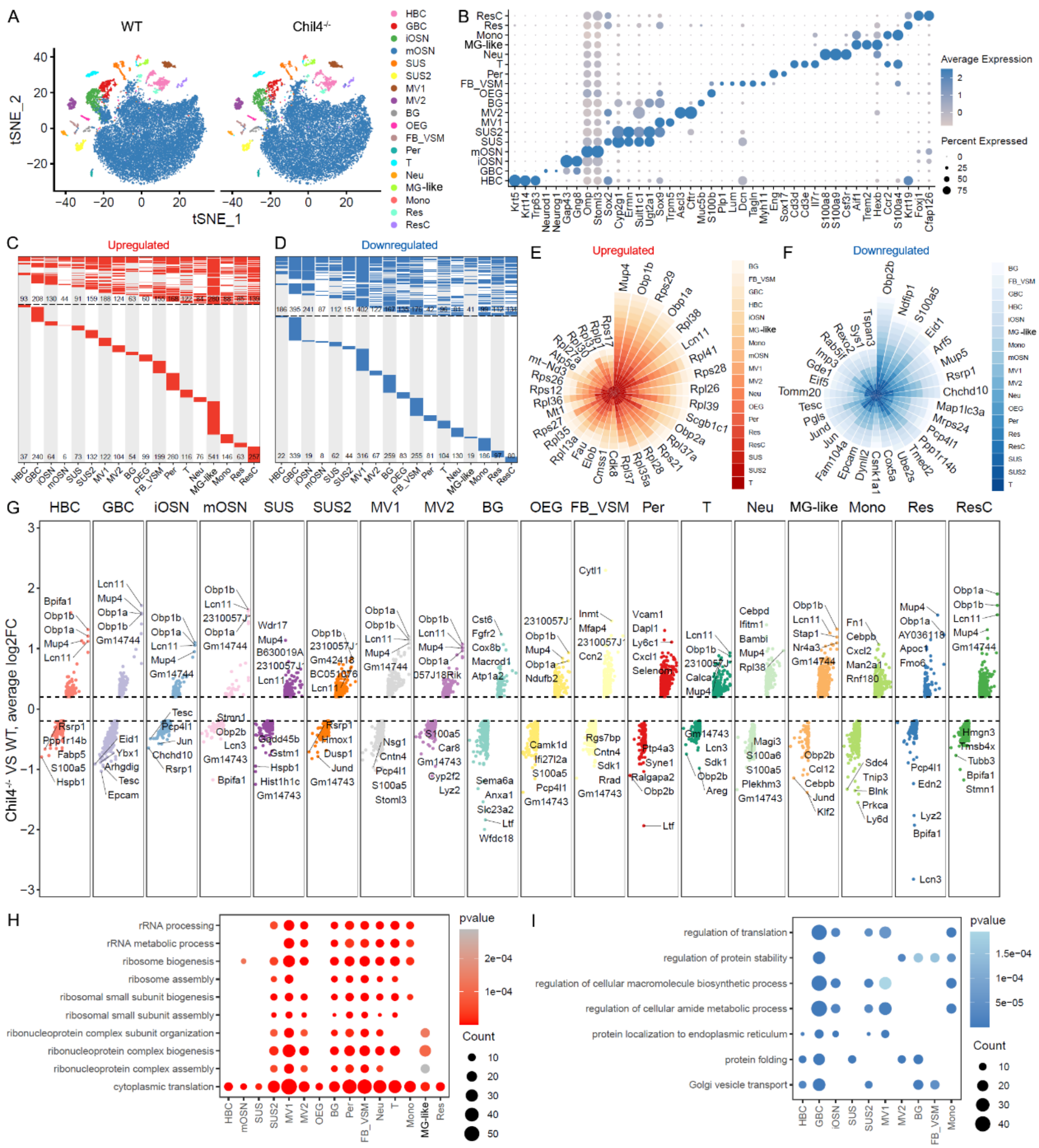

Figure S5. Differentially expressed gene in various cell subtypes of the Chil4$^{-/-}$ OE. (A) tSNE plot showing cell subtypes in the WT and Chil4$^{-/-}$ OE. (B) Dot plot showing molecular markers in different cell types. (C, D) Heatmaps of upregulated (C) and downregulated genes (D) globally or specifically expressed in different cell types of Chil4$^{-/-}$ OE compared to WT tissue. (E, F) Plots of single upregulated (E) and downregulated genes (F) in multiple cell types of Chil4$^{-/-}$ OE compared to WT tissue. (G) Five most upregulated and downregulated genes in various cell types of Chil4$^{-/-}$ OE compared to WT tissue. (H, I) GO enrichment analysis of upregulated (H) and downregulated (I) genes in the Chil4$^{-/-}$ OE.

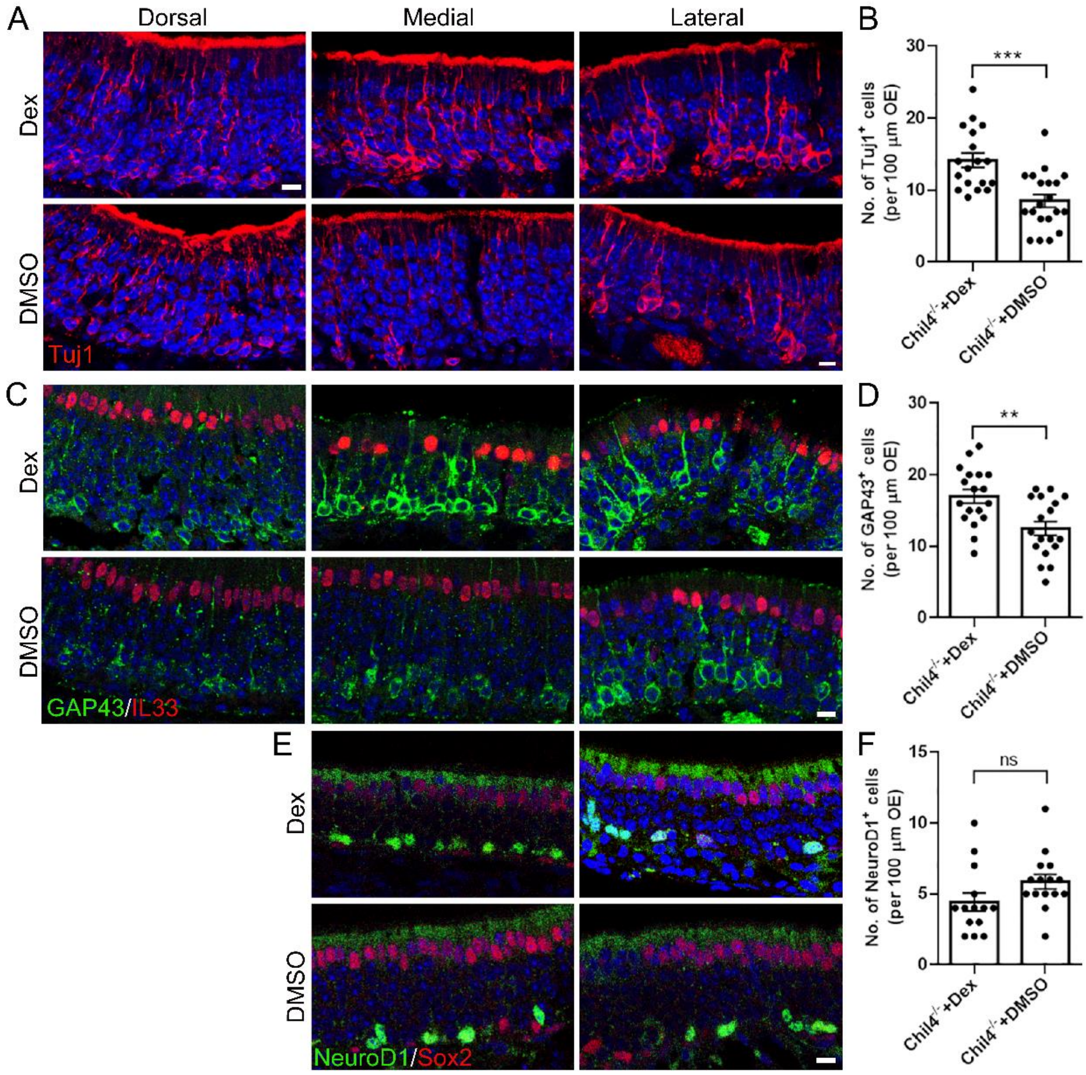

Figure S6. Dexamethasone (Dex) recovers generation of iOSNs in the Chil4$^{-/-}$ OE. Confocal images of Tuj1^{+} iOSNs (A), IL33^{+} sustentacular cells (C), GAP43^{+} iOSNs (C), Sox2^{+} sustentacular cells (E), NeuroD1^{+} GBCs (E) in the OE of Chil4$^{-/-}$ mice receiving Dex or DMSO. (B, D, F) Quantification of Tuj1^{+} iOSNs (B), GAP43^{+} iOSNs (D), NeuroD1^{+} GBCs (F) in the OE of Chil4$^{-/-}$ mice receiving Dex or DMSO. n = 18 and 20 sections for Dex and DMSO group in (B), n = 18 and 18 sections in (D), n = 14 and 15 sections in (F). The statistical significance was determined by unpaired t test. ns, not significant, $^{**}p < 0.01$, $^{***}p < 0.001$. Scale bars: 10 µm.
